# Cholera- and Anthrax-Like Toxins Are among Several New ADP-Ribosyltransferases

**DOI:** 10.1371/journal.pcbi.1001029

**Published:** 2010-12-09

**Authors:** Robert J. Fieldhouse, Zachari Turgeon, Dawn White, A. Rod Merrill

**Affiliations:** Department of Molecular and Cellular Biology, University of Guelph, Guelph, Ontario, Canada; Stockholm University, Sweden

## Abstract

Chelt, a cholera-like toxin from *Vibrio cholerae*, and Certhrax, an anthrax-like toxin from *Bacillus cereus*, are among six new bacterial protein toxins we identified and characterized using *in silico* and cell-based techniques. We also uncovered medically relevant toxins from *Mycobacterium avium* and *Enterococcus faecalis*. We found agriculturally relevant toxins in *Photorhabdus luminescens* and *Vibrio splendidus*. These toxins belong to the ADP-ribosyltransferase family that has conserved structure despite low sequence identity. Therefore, our search for new toxins combined fold recognition with rules for filtering sequences – including a primary sequence pattern – to reduce reliance on sequence identity and identify toxins using structure. We used computers to build models and analyzed each new toxin to understand features including: structure, secretion, cell entry, activation, NAD^+^ substrate binding, intracellular target binding and the reaction mechanism. We confirmed activity using a yeast growth test. In this era where an expanding protein structure library complements abundant protein sequence data – and we need high-throughput validation – our approach provides insight into the newest toxin ADP-ribosyltransferases.

## Author Summary

Computer tools helped us uncover and understand potent protein toxins that empower bacterial pathogens against plants, animals and man. These toxins are potential drug targets and researchers can use them to make vaccines. New toxin knowledge aids the long-term goal of finding alternatives to antibiotics, to which pathogens are becoming more resistant. The toxins share similar structure despite low sequence identity, so our search links sequence and structure features. We present a ranked list and computational characterization of six new toxins combined with cell-based tests.

## Introduction

Sequence data from over 6,500 genome projects is available through the Genomes OnLine Database [1] and more than 60,000 protein structures are in the Protein Data Bank (PDB). While these sequences represent large diversity, a limited number of possible folds – estimated at 1,700 [2] – helps researchers organize the sequences by structure. A single fold performs a limited number of functions, between 1.2 and 1.8 on average [3]. Therefore, structure knowledge helps pinpoint function. Researchers are combining sequence and structure data to expand protein families such as the mono-ADP-ribosyltransferase (mART) protein toxins that participate in human diseases including diphtheria, cholera and whooping cough [4].

ADP-ribosylation is a post-translational modification that plays a role in many settings [5]. ADP-ribosyltransferases (ADPRTs) bind NAD^+^ and covalently transfer a single or poly ADP-ribose to a macromolecule target, usually protein, changing its activity. Many prokaryotic ADPRT toxins damage host cells by mono-ADP-ribosylating intracellular targets. G-proteins are common targets including: eukaryotic elongation factor 2 (ADP-ribosylation halts protein synthesis), elongation factor thermo unstable, Ras, Rho (ADP-ribosylation locks Rho GTPase in the GDP-bound state and disaggregates the actin cytoskeleton) and Gs-α (ADP-ribosylation interrupts signal transduction). Other targets include actin (ADP-ribosylation inhibits actin polymerization) [6]; kinase regulators (ADP-ribosylation inhibits phagocytosis) [7] and RNA-recognition motifs (ADP-ribosylation alters the transcriptome and weakens immunity) [8].

Researchers use ADPRT toxins to develop vaccines [9], as drug targets, to kill cancer cells [10], as stent coatings to prevent restenosis after angioplasty [11], as insecticides, to deliver foreign proteins into cells using toxin receptor-binding and membrane translocation domains, to study cell biology [12], [13], to understand the ADP-ribosylation reaction and to identify biosecurity risks.

ADPRTs occur in viruses, prokaryotes, archaea and eukaryotes. Genomes acquire them through horizontal gene transfer [14]–[17]. Several authors have reviewed the prokaryotic ADPRT family [6], [18], [19]. Examples include *Pseudomonas aeruginosa* exoenzyme S (ExoS), *Vibrio cholerae* cholera toxin (CT), *Bordetella pertussis* pertussis toxin (PT) and *Corynebacterium diphtheriae* diphtheria toxin (DT). Toxic ADPRTs are divided into the CT and DT groups to better organize the family. We focus on the CT group, which we divide into the ExoS-like, C2-like, C3-like and CT-PT-like toxins.

**Figure 1:**
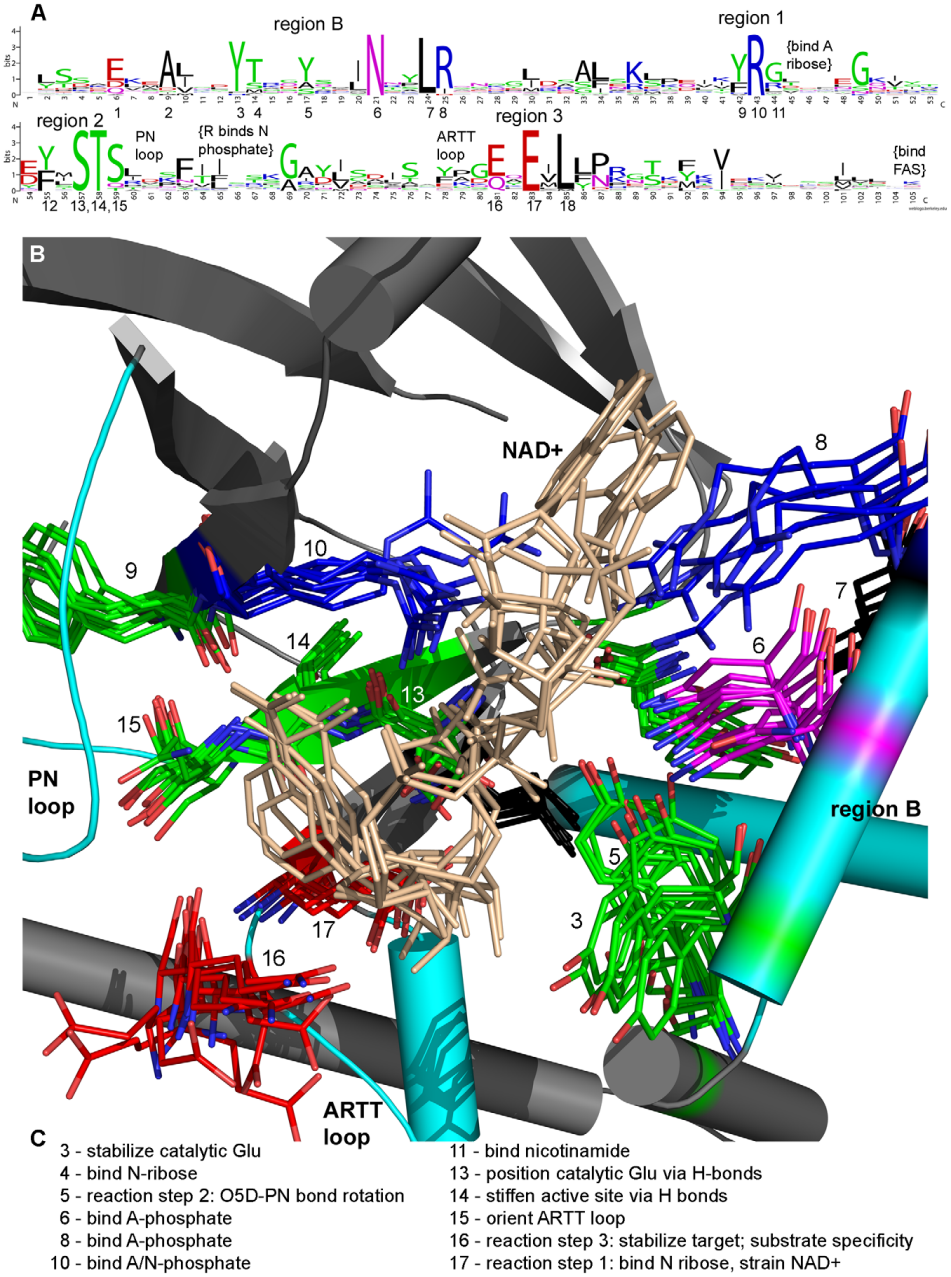
Sequence-structure-function relationships in ExoS-like, C2-like and C3-like toxins. (A) The curated sequence alignment presented in LOGO-format. The largest residues are important for catalysis and perhaps also folding. Difficult-to-read text is unimportant. (B) Multiple structure alignment of the active site showing structural position of the conserved residues. PDB IDs: Iota (1GIQ), Art2.2 (1OG3), C3stau2 (1OJZ), Vip2 (1QS1), C3bot2 (1R45), C3bot1 (2A9K), SpvB (2GWL), C2-I (2J3X), CdtA (2WN7), C3lim (3BW8). Important residues have a relatively constant position. NAD^+^ position is more variable toward the adenine end of the dinucleotide. (C) Functional relevance of active site residues [6]. Numbers not listed imply a role not yet assigned.

**Figure 2:**
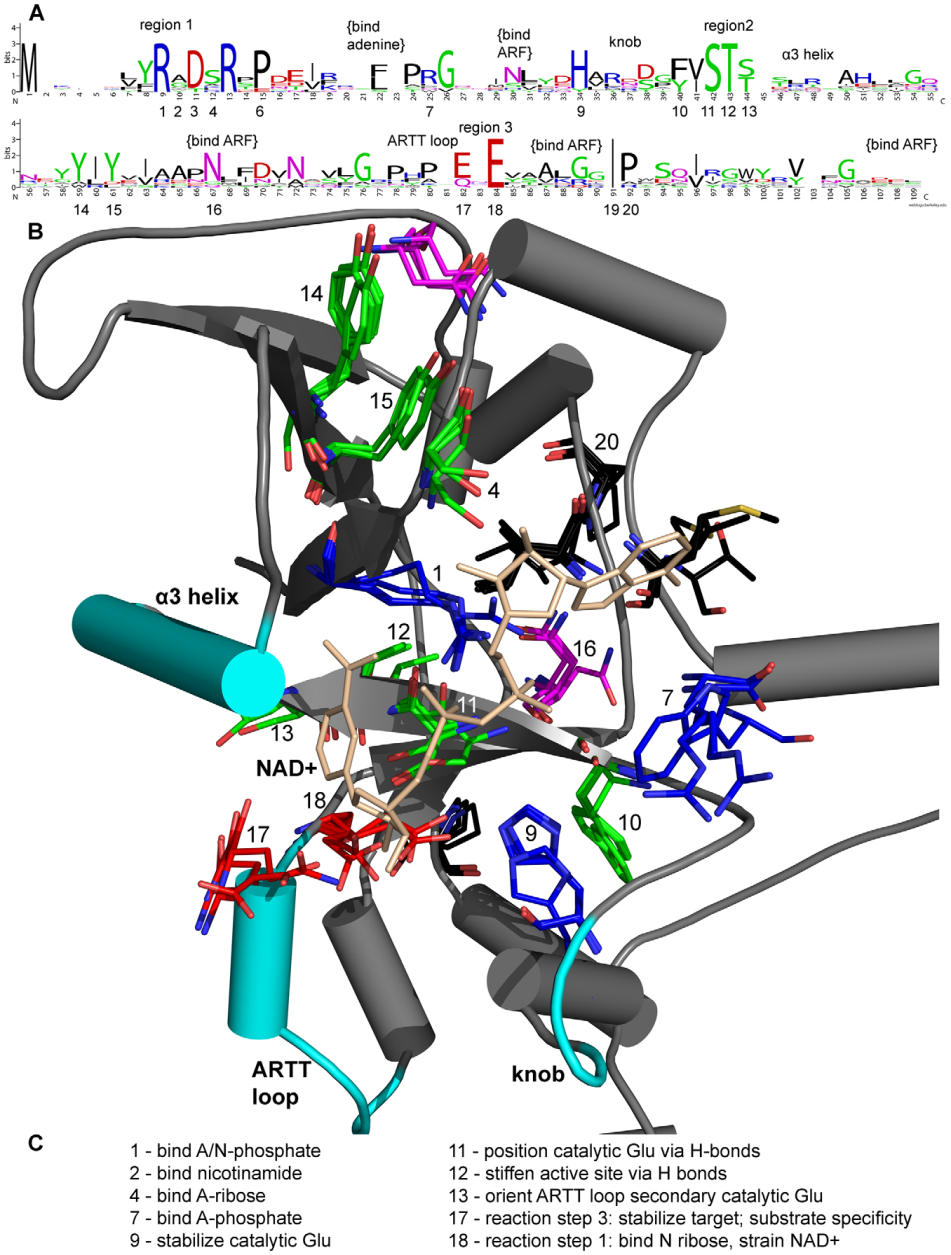
Sequence-structure-function relationships in CT-PT-like toxins. (A) The curated sequence alignment presented in LOGO-format. The largest residues are important for catalysis and perhaps also folding. Difficult-to-read text is unimportant. (B) Multiple structure alignment of the active site showing structure conservation of these residues. PDB IDs: CT (1S5D), LT-IIB (1TII), LT-A (1LTS), PT (1BCP), CT (2A5F). Little variation exists in important residue positions. (C) Functional relevance of active site residues [6]. Numbers not listed imply a role not yet assigned.

CT group primary sequences are related through a specific structure-linked pattern (Figures 1 and 2) [20]. The ADPRT pattern, updated from previous reports [4], [21] and written as a regular expression is:
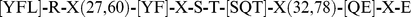



The toxin catalytic domain consists of several regions. We describe them here going from the N- to C-terminus using previously introduced nomenclature [20], [22]. Region A (not shown) is sometimes present and recognizes substrate, when ExoT recognizes Crk, for example. Its recognition of ExoT targets is an exception rather than a general rule for ADPRTs. Except for the CT-PT-like subgroup, region B – an active site loop flanked by two helices – appears early in the toxin sequence. It stabilizes the “catalytic” Glu, binds the nicotinamide ribose (N-ribose) and the adenine phosphate (A-phosphate). It also stabilizes the target substrate and helps specific bonds rotate during the ADPRT reaction, in turn, helping to bring the nucleophile and electrophile together for reaction. (The CT-PT-like subgroup lacks region B and instead has a knob region that precedes region 2; these might function interchangeably.) Region 1 is at the end of a β-sheet, with sequence pattern [YFL]RX. It is important for binding A-phosphate, nicotinamide phosphate (N-phosphate), nicotinamide, adenine ribose (A-ribose) and the target substrate. Region F (not shown) follows region 1 and sometimes recognizes substrate. The region 2 (STS motif) follows on a β-sheet with sequence pattern [YF]-X-S-T-[SQT]. It binds adenine, positions the “catalytic” Glu, orients the ADP-ribosyl-turn-turn (ARTT) loop and maintains active site integrity. The phosphate-nicotinamide (PN) loop (also known as region E) is immediately after the STS motif. It interacts with the target and binds N-phosphate. Menetrey *et al.* suggested the PN loop is flexible and implicated it in locking the nicotinamide in place during the reaction [23]. Region 3 (also known as region C) consists of the ARTT loop leading into the β-sheet with pattern [QE]-X-E. It recognizes and stabilizes the target and binds the N-ribose to create a strained NAD^+^ conformation. The ARTT loop is plastic, having both “in” and “out” forms that might aid substrate recognition [23]. The FAS region (also known as region D, not shown) mediates activator binding when present [6], [22], [24], [25].

Researchers have long debated the ADPRT reaction details. Some suggest an S_N_2 mechanism [26], [27], but many now favor the S_N_1 mechanism [28]–[32]. Tsuge *et al.* recently devised a specific version of this mechanism for iota toxin, which we follow closely in this work [33], [34]. The reaction follows three steps: the toxin cleaves nicotinamide to form an oxacarbenium ion, the oxacarbenium O_5D_-P_N_ bond rotates to relieve strain and forms a second ionic intermediate. (The electrophile and nucleophile might migrate by an unknown mechanism to further reduce the distance between them.) Finally, the target makes a nucleophilic attack on the second ionic intermediate. The S_N_1mechansim – believed widely applicable to CT group toxins – is a template for new toxins given the historical structure similarity and consistent NAD^+^ conformation in the active site as shown in Figures 1 and 2.

Quaternary structure for the toxins is wide-ranging. Several combinations exist for toxin domains (A) and receptor binding or membrane translocation domains (B). The B domains have diverse structures and functions and exist as fusions or separate polypeptides. Various formats include: A-only, two-domain AB (single polypeptide), three-domain AB (single polypeptide) and AB_5_ (multiple polypeptides). C3-like toxins are A-only. ExoS-like toxins have toxic A-domains and are often paired with Rho GTPase activating protein (RhoGAP), which are not true B domains. C2-like toxins are AB toxins that contain B domains that are structural duplicates of the A domain. These B domains are not toxins; they bind proteins that are similar to anthrax protective antigen (PA) including Vip1, C2-II and Iota Ib [35], [36]. DT group toxins are three-domain, single polypeptide AB toxins where the B domain contains both a receptor-binding and a membrane-translocation domain. The CT-PT-like toxins are AB_5_ and have B domains that form a receptor-binding pentamer [37].

Low overall sequence identity hampers conventional sequence-based homology searches [17], [20], [38]–[40]. One challenge – key to filling gaps in the toxin family – is to link new sequences and known toxins. Depending only on amino acid sequence alignment techniques to discover new toxins is imprudent. Instead the trend is to use more structure information in the search because many primary sequences produce the same fold [41]. Researchers can then link these sequences through fold recognition [42].

Otto *et al.* used PSI-BLAST to identify new ADPRT toxins, including SpvB from *Salmonella enterica* [14]. More recently a similar strategy yielded 20 potential new toxins [15]. This led to interesting examples later characterized including: CARDS toxin from *Mycoplasma pneumonia* [43], SpyA from *Streptococcus pyogenes* [44] and HopU1 from *Pseudomonas syringae* [8].

PSI-BLAST is a classic way to expand protein families, but it has limits. For example, unrelated sequences often “capture” the search. Also, nearly a decade has passed since Pallen *et al.* released the last detailed data mining results for the toxin family [15]. The sequence and structure databases – and remote homolog detection tools – have advanced during this time. Masignani *et al.* proposed that a match between the conserved ADPRT pattern with corresponding secondary structure is one way to reduce dependence on sequence identity. The pattern helps ensure function and reduces the total sequence set to a smaller subset for screening; secondary structure prediction ensures that key active site parts are present [17].

Our contribution is to expand ADPRT toxin family using a new approach. The difference is that we use fold-recognition searches extensively rather than relying on PSI-BLAST or secondary structure prediction. Our genomic data mining combines pattern- and structure-based searches. A bioinformatics toolset allows us to discover new toxins, classify and rank them and assess their structure and function. Often, data mining studies simply present a table of hits with aligned sequences, but do not interpret or analyze those hits in detail. Our aim – rather than to explicitly confirm the roles of the six proteins, 15 domains, 18 loops and 120+ residues discussed – is to develop a theoretical framework for understanding new toxins, based on 100s–1000s of jobs per sequence. We intend our *in silico* approach to guide and complement – rather than replace – follow-up *in vitro* and *in vivo* studies. Here, we extract features and patterns from known ADPRT toxins and explain how they fit new toxins. We use *in silico* methods to probe structure, secretion, cell entry, activation, NAD^+^ substrate binding, intracellular target binding and reaction mechanism.

A computer approach is fitting for several reasons. Such an environment is a safe way to study new toxins. Challenges in cloning, expressing, purifying and crystallizing often prevent *in vitro* characterization. Also, ADPRTs are abundant within bacterial genomes and researchers make the sequences available faster than we can conduct biochemical studies. New toxins might play a role in current outbreaks and are also excellent drug targets against antibiotic resistance. Our new study design expands the family by ∼15% (from 36 to 42 toxins).

Cell-based validation complements our *in silico* approach. We use *Saccharomyces cerevisiae* as a model host to study toxin effects. Increasingly, researchers are turning to yeast to study bacterial toxins. Yeast are easy to grow, have well-characterized genetics and are conserved with mammals in cellular processes including: DNA and RNA metabolism, signalling, cytoskeletal dynamics, vesicle trafficking, cell cycle control and programmed cell death [45]–[47]. We place the toxin genes under the control of a copper-inducible promoter to test putative toxins for ADP-ribosyltransferase activity in live cells [48]. A growth-defective phenotype clearly shows toxicity. Substitutions to catalytic signature residues confirms ADP-ribosyltransferase activity causes the toxicity. Indeed, pairing *in silico* and cell-based studies helps identify and characterize new ADPRT toxins.

## Results/Discussion

### Data mining for new ADPRT toxins

We searched fold-recognition databases – including Pfam 24.0 [49], Gene3D 9.1.0 [50] and SUPERFAMILY 1.73 [51] – using SCOP and CATH codes of known toxins. These strategies relate sequences with profiles. We also used a sensitive profile-profile based search strategy, HHsenser 2.13.5 [52]. We combined the results from our various searches and filtered them by successively applying exclusions to discover new ADPRT toxins. First, we had 2106 hits. We kept only bacterial hits (lost 1222) from pathogens (lost 445) that tested positive for secretion (lost 95), had the conserved ADPRT pattern (lost 218) and had less than 50% identity to a known toxin (lost 87). This left 39 hits. We reduced them to 29 by clustering at the 50% identity level. We removed one more sequence on the basis of genetic context (a hydrolase gene was next to the toxin gene, suggesting possible de-ADP-ribosylation reactions). This left 28 sequences. Of these, we found 15 from Pfam, Gene3D and HHsenser; eight from both Gene3D and HHsenser; four from HHsenser only; and one from both Pfam and Gene3D. We chose five of the 28 sequences to analyze more thoroughly. We also present our analysis of TccC5, a toxin we previously proposed [4] that Lang *et al.* biochemically characterized during this writing [53].

We count 36 known ADPRT toxins (see [4] for a recent table and note that researchers recently characterized several [54]–[57]). The six described in this writing bring the total to 42 distinct ADPRT toxins that generally have identity <50% unless the species or domain organization is different. We may want to remove the pattern constraint in the future and further expand the toxin pattern. Here, we prefer higher accuracy at the risk of removing some true ADPRT toxins from our list. Five of the six toxins described appear in a simple protein-protein BLAST search. But identity is typically low enough that many false hits appear as well. This makes the simple BLAST search ineffective. Randomly created sequences, for example, regularly return BLAST hits at ∼25% identity. (For example, we tried 10 BLAST searches using 200-residue random sequences with average Swiss-Prot amino acid composition. We received top hits of average length 99 and having 29% identity to a natural protein.)

We ranked the toxin candidates by relevance signalled by ISI Web of Knowledge hits to the species name (Table 1). As well, we list the fold prediction strength given by J_3D-jury_ and catalytic domain novelty suggested by sequence identity to the nearest known toxin. 3D-jury accepts models from various servers and makes pair-wise comparisons. Each pair gets a similarity score that equals the total number of Cα atom pairs within 3.5Å after overlap. The final score is the sum of the similarity scores divided by the number of pairs considered plus one. A higher J_3D-jury_ implies a stronger prediction. The closest toxin relative to a newly predicted toxin indicates the new toxin's novelty and aids function prediction. Identity to a known toxin ranges from 25% to 60%. We show predictions for the toxins in Table 2.

**Table 1:** New ADPRT toxin ranking.

Rank	Name	Species	Accession	Interest	I^a^	J^b^	Structure match	N^c^	Sequence match
1	Chelt	*V. cholerae* MZO-3	A2PU44	Cholera, food poisoning	12,458	178.12	LT-A (1LT4)	60	LT-IIA, LTII-B
2	Certhrax	*B. cereus* G9241 [269801]	Q4MV79	Inhalation anthrax, food poisoning	11,529	217.25	Anthrax lethal factor (1J7N)	34	CdtA
3	Mav toxin	*M. avium* (strain 104)	A0QLI5	Respiratory infection, tuberculosis-like pulmonary infection	11,289	125.75	C3bot1 (2BOV)	30	HopU1, AexT
4	EFV toxin	*E. faecalis*	Q838U8	Urinary infection, bacteremia, endocarditis	10,422	158.12	C2-I (2J3Z)	29	C3lim
5	TccC5	*P. luminescens* (laumondii)	Q7N7Y7	Toxemia, septicaemia	556	49	C3bot2 (1R45)	25	SpvB
6	Vis toxin	*V. splendidus* 12B01	A3UNN4	Vibriosis	241	129.4	Iota (1GIQ)	28	C2-I, HopU1

a I = ISI Web of Knowledge hits to species name.

b J = J-score from 3D-jury consensus fold recognition.

c N = percent identity (catalytic core) to known ADPRT.

**Table 2:** New ADPRT toxin features.

Name	Length	Domains^a^	Secretion^b^	P_soluble_ c	Crystallization^d^	Target^e^
Chelt	601	Ia (∼1–179, toxin); Ib(∼180–240); II (∼241–601, lectin-like)	SP (18|19)	0.141	Amenable	G_αs_ R201
Certhrax	476	I (∼1–241, PA binding); II (∼242–476, toxin)	0.706	0.519	High scoring	Asn, Gln or Cys?
Mav toxin	825	I (∼1–96, ESAT6); II (∼97–439, pore forming?); III (∼440–675, disordered); IV (∼675–825, toxin)	0.822	0.332	Recalcitrant	Arg?
EFV toxin	487	I (∼1–309, needle/pore forming?); II (∼310–487, toxin)	0.731	0.258	Recalcitrant	Arg?
TccC5	938	I (∼1–341, β-propeller); II (∼342–675, β-propeller); III (∼676–738, helical); IV (∼739–938, toxin)	0.936	0.599	Amenable	RhoA Q61
Vis toxin	249	I (1–249, toxin)	SP (18|19)	0.611	Amenable	Arg?

a Predicted by DOMAC, Ginzu or sliding-window fold recognition.

b SecretomeP scores >0.5 suggest secretion without specific signal peptide; “SP” indicates secretion signal peptide detected by SignalP.

c Probability of solubility by PROSO.

d ParCrys crystallization propensity.

e Expected target amino acid (refer to main text for more information).

**Figure 3:**
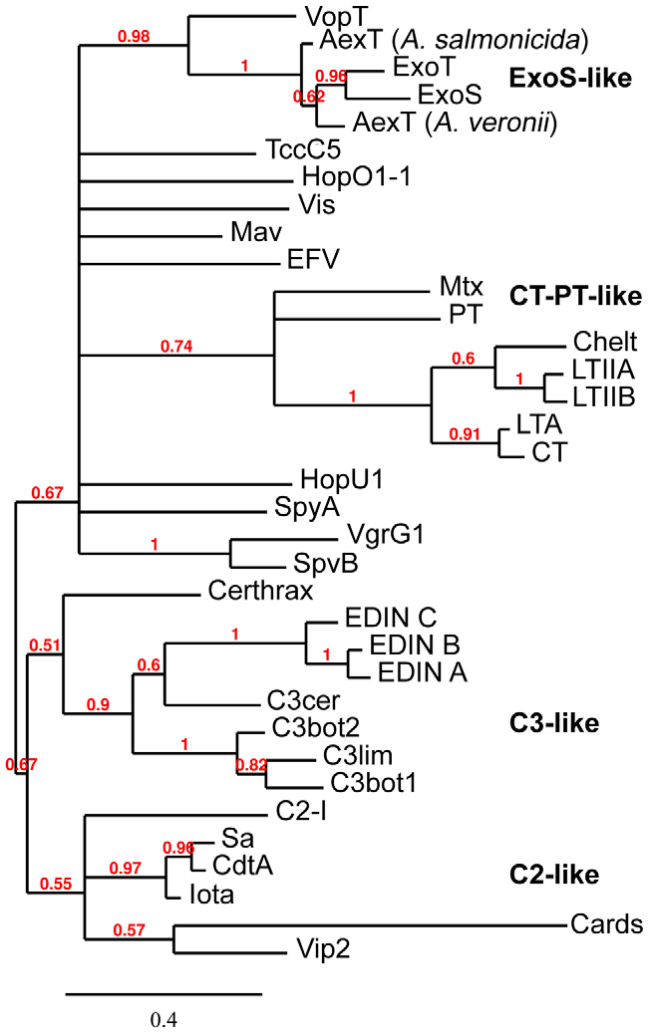
Phylogenetic tree shows relationships between toxins. This phylogenetic tree reveals four known CT ADPRT toxin subgroups: ExoS-like, C2-like (includes the SpvB-like toxins), C3-like and CT-PT-like (includes cholera and pertussis toxins). We built the tree using an alignment of all ADPRT toxins and MrBayes, which uses Bayesian inference and a Markov Chain Monte Carlo hill-climbing algorithm to arrive at a near-optimal tree [96]. We annotated the branches with bootstrap values. (CARDS toxin is normally considered part of the CT-PT-subgroup; it is in an unusual position in this tree.)

Aligned sequences of known and new CT group toxins are critical to further studies (Figures S1 and S2 in Text S1). We removed positions with gaps and represented the alignment in LOGO format for the ExoS-like, C2-like, C3-like subgroups (Figure 1) and the CT-PT-like subgroup (Figure 2). Also, we correlated critical residues with previous X-ray structures and function information. We used an alignment that contained all CT group toxins to build a phylogenetic tree that groups known and new toxins into subgroups, shown in Figure 3. We use this tree to show relationships between the toxins independent of any specific evolutionary pathway. Such a pathway is difficult or impossible to deduce because of horizontal, rather than vertical, gene transfer. We did not include eukaryotic ARTs in our tree because they are not within this paper's scope. But, they often group well with C3-like toxins, and many eukaryotic PARPs group with the DT group toxins. Also, we calculated a pair-wise identity matrix (Table S1 in Text S1), revealing identity between known and new CT group toxins. We invite readers to skip to the species or toxin of most interest; each one is described independently.

### *V. cholerae* Chelt: A new cholera toxin with likely different cell-entry machinery

**Figure 4:**
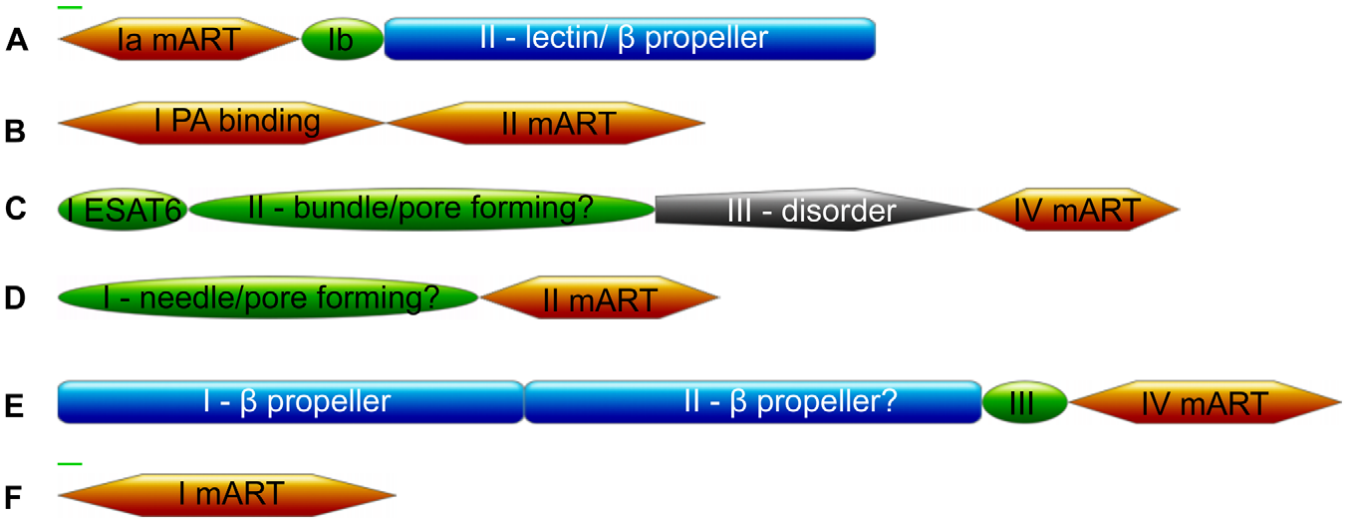
Toxin domain combinations. Domain combinations in the new ADPRT toxins based on DOMAC, Ginzu and sliding-window fold recognition data. Mainly α-helix (green oval), mainly β-sheet (blue rectangle), α/β or α+β alpha-beta mixtures (orange), mainly loop or disordered (grey). We mark secretion signal peptides with a green line. (A) Chelt (B) Certhrax (C) Mav toxin (D) EFV toxin (E) TccC5 (F) Vis.

**Figure 5:**
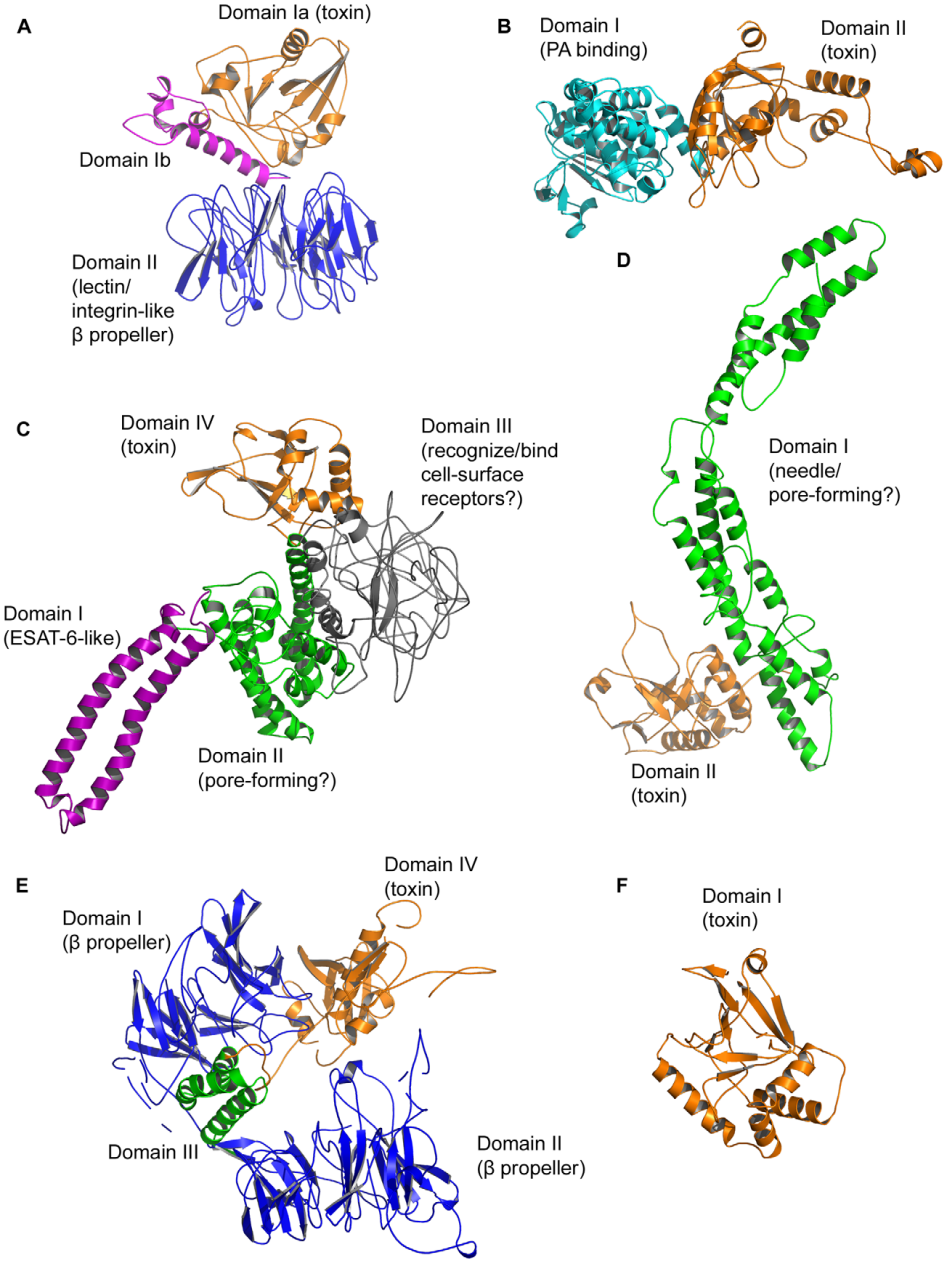
Full-length structure models. Full-length models, produced using templates for individual domains and, where necessary, docking the domains together. The goal is to understand overall features such as secondary and super-secondary structure, topology and the possible multi-domain enzyme structure. We do not imply any specific domain orientations nor make claims about the exact nature of the structure, especially regarding embellishments to each domain's core fold. We modeled the new ADPRT toxins using the I-TASSER server [121] and also MODELLER with suitable templates. (A) Chelt (B) Certhrax (C) Mav toxin (D) EFV toxin (E) TccC5 (F) Vis. Quality scores are in Tables S2 and S3 in Text S1.

*V. cholerae* produces cholera and cholix toxins [4]. Chelt (UniProt A2PU44) is, to our knowledge, the third ADPRT toxin identified in *V. cholerae*, the bacterium responsible for cholera outbreaks and food poisoning. The genome sequence of *V. cholerae* strain MZO-3 serogroup O37, isolated from a patient in Bangladesh (Heidelberg, J. and Sebastian, Y., 2007, Annotation of *Vibrio cholerae* MZO-3, TIGR) encodes Chelt. It is specific to this strain. Chelt GC content is 14% lower than the overall genome (34% vs. 48%); also, a transposase gene immediately follows the Chelt gene, indicating horizontal gene transfer typical of the ADPRT toxins. Chelt is a 601-residue, 69 kDa protein. It has a secretion signal (∼1–18), followed by toxin domain Ia (∼19–179) and Ib (∼180–240) and a presumed cell-binding domain II (∼241–601) (Figures 4A and 5A).

Chelt is unusual in that it has a second domain attached to the catalytic domain (Figure S3 in Text S1). Because the genome does not obviously encode a B-domain pentamer, domain II could fulfill that role. After secretion, Chelt likely uses it to bind to the cell surface. Domain II has significant structure similarity to *Psathyrella velutina* lectin (PDB 2BWR; 15% identity; J_3d-jury_ = 152; an easy target for the Local Meta-Threading-Server LOMETS, which provides this high-confidence match). Weaker similarities also exist to human integrin αVβ3 (PDB 2VDR; 11% identity; an easy target for LOMETS, which provides this high-confidence match). Prokaryotic lectins allow differential eukaryotic cell recognition. Indeed bacterial lectins can mimic eukaryotic adhesion motifs [58]. Structurally, the domain is a seven-bladed β-propeller (SCOP b.69.8, CATH 2.130.10), with each blade containing seven four-stranded β-sheet motifs that meander. The lectin suggests a role in sugar and Ca^2+^, or possibly Mg^2+^, binding and perhaps even integrin mimicry. Chelt is reminiscent of ricin toxin from the castor bean. Ricin is a two-domain toxin that contains both a lectin for binding the cell-surface galactosyl residues for cell-entry and a second domain that causes cell death [59].

Domain I, the catalytic domain, is 60% identical to LT-A from *Escherichia coli*. This toxin clearly fits into the G_αs_–targeting CT-PT-like subgroup because sequence identity to LT-A is so high. Fold recognition returned a match to LT-A (PDB 1LT4, J_3D-jury_ = 178) and our model against this template was also high quality. The Chelt catalytic domain adopts an α+β ADP-ribosylation fold consisting of anti-parallel β-sheets and having separate α and β regions.

Chelt must likely be activated by reduction of a disulfide bond between Chelt C205 and C220; cleavage at or near I215 (details are unclear due to a four amino acid deletion compared to LT-A between H214 and I215); and interaction with an ADP-ribosylating factor, perhaps ARF3, in the Chelt regions ∼45–57, ∼109–113, ∼134–141 and ∼167–182 (Figure S3 in Text S1).

**Figure 6:**
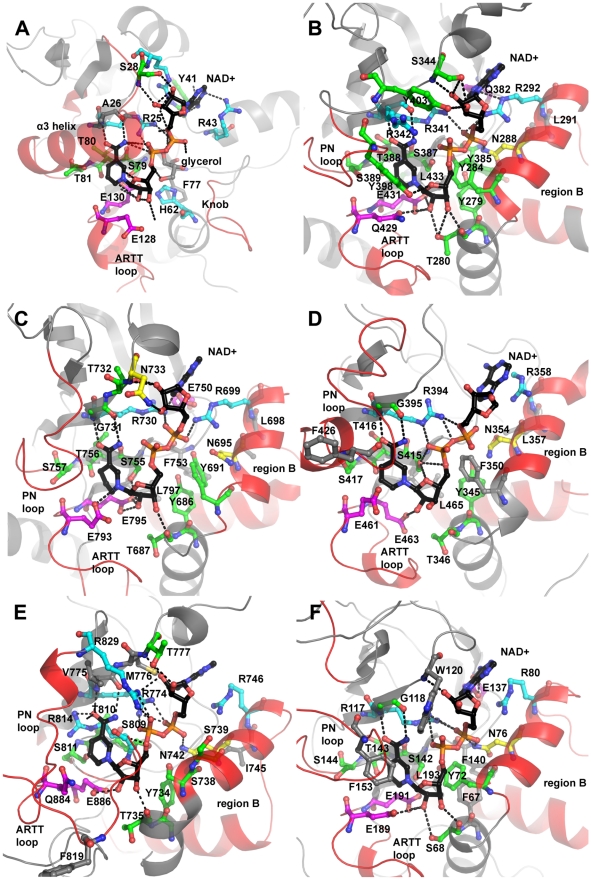
Active site structure models with NAD^+^ bound reveal important residues. NAD^+^-bound active-site models, developed using homology-based transfer. We used them to help reveal important residues and help understand plausible NAD^+^-binding modes and reaction mechanisms. These active-site models contain NAD^+^ fit into the active site. We do not intend to imply specific loop conformations or the nature of embellishments to the core fold. We built the models using MODELLER. Modeled active sites include: (A) Chelt (B) Certhrax (C) Mav toxin (D) EFV toxin (E) TccC5 (F) Vis toxin. Quality scores are in Tables S2 and S3 in Text S1.

**Figure 7:**
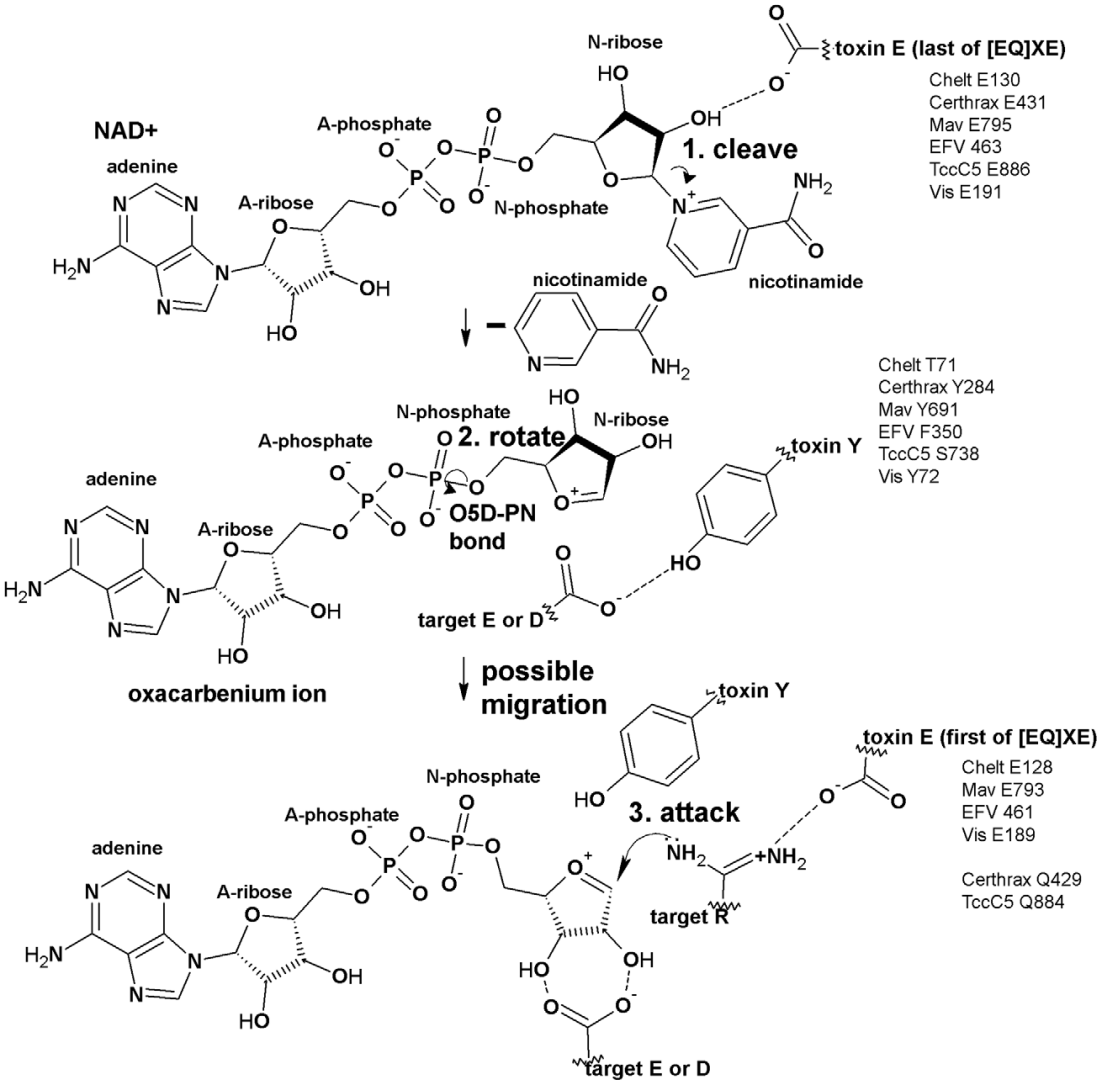
Proposed mechanisms for the new toxins by homology-based transfer. The S_N_1 alleviated-strain mechanism, developed for Iota toxin, is likely widely applicable throughout the CT group ADPRTs [34], given high structure similarity and consistent NAD^+^ conformation in the active site. Therefore, we use a 3DLOGO-based method to propose a homology-based mechanism for the new ADPRTs. First, the universally conserved region 3 catalytic Glu (which H-bonds to the N-ribose) and the universally conserved region 1 Arg (which creates phosphate electrostatic interactions) hold the NAD^+^ in a conformation that favors oxacarbenium ion formation. Then, we invoke a Phe as well as the Tyr that induces a rotation of the oxacarbenium ion about the O_5D_-P_N_ bond of the N-ribose to relieve the strained NAD^+^ conformation and help reduce the nucleophile-electrophile distance. (Previous work has shown the Tyr to Phe substitution in Iota toxin is still active [34].) The electrophile and nucleophile may migrate by an unknown mechanism that further reduces the distance between them. Finally, a target Glu or Asp stabilizes the N-ribose, the region 3 Glu or Gln stabilizes the target Arg, Asn or Cys; Asn, Gln or Cys attacks the oxacarbenium ion, for region 3 QXE toxins, or an Arg attacks the oxacarbenium ion for region 3 EXE toxins.

We propose a likely mode of NAD^+^ binding, target binding and ADP-ribosylation based on alignment data and our modeling experiments. Once activated, Chelt binds NAD^+^ through hydrogen bonds, hydrophobic interactions and aromatic interactions (Figure 6A, Figure S4 in Text S1, Table 3). We propose these H-bonds: Y41 binds to adenine, S28 binds to A-ribose, R43 binds to A-phosphate, R25 binds to A- or N-phosphate, E130 binds to N-ribose and A26 binds to nicotinamide. Chelt recognizes G_αs_ using the knob (∼66–71), the α3 helical region (∼82–99) and the ARTT loop (∼104–129) (Table 4). The ARTT loop might plastically rearrange between the in and out conformation during this process. Anchor residues S123 and Q127 in the second part of the loop may act as hinges to reposition H125 to interact with G_αs_. We propose an S_N_1 alleviated-strain mechanism (Figure 7). First, E130 H-bonds to the N-ribose while phosphate electrostatic interactions hold the NAD^+^ in a conformation that favors oxacarbenium ion formation. The reaction's progress is unclear. T71 might induce a rotation about the O_5D_-P_N_ bond of the oxacarbenium ion to reduce the nucleophile-electrophile distance. A G_αs_ Glu or Asp stabilizes N-ribose, E128 stabilizes G_αs_ R201 and G_αs_ R201 attacks the oxacarbenium ion. Several residues hold the active site in place including: Chelt S79, which H-bonds to E130; T80, which stiffens the active site through H-bonding to a nearby β-sheet and T81, which orients the ARTT loop and E128. Hydrophobic interactions with NAD^+^ involve D27, R29, P42, I90, I94 and L95. Also, H62 stabilizes E130.

**Table 3:** Residues important for NAD^+^ binding and reaction.

Region	Role	Iota toxin comparison (wt = 100)^a^	Chelt	Certhrax	Mav toxin	EFV toxin	TccC5	Vis toxin
	rearranges upon target binding, stabilizes last E of [QE]XE, may bind “vital N”, may reposition N-ribose ring	Y246	H62	Y279	Y686	Y345	Y734	F67
	binds N-ribose			T280	T687	T346	T735	S68
	reaction step 2, rearranges on target binding, aids rotation of O_5D_-P_N_ bond, may “catch” oxacarbenium ion before transfer	Y251A: 0; Y251F: 90	T71	Y284	Y691	F350	(S738, S739}	Y72
Vital N	binds A-phosphate	N255		N288	N695	N354	N742	N76
	may position “vital N”	L258		L291	L698	L357	I745	L79
region B active site loop	binds A-phosphate		R43	R292	R699	R358	R746	R80
region 1	unclear	Y294	Y24	Y340	V729	Y393	Y773	Y116
region 1 R	binds A/N-phosphate	R295A: 0; R→K: 1	R25	R341	R730	R394	R774	R117
region 1 R	binds nicotinamide	R296; (backbone H bond)	A26	R342	G731	G395	V775	G118
	binds A-ribose^b^ (2 residues)	E301A: 0	S28	∼343–348 (S344}	∼732–737 (T732, N733 }	∼396–400 (N399, E400}	∼776–780; (T777}	∼119–123 (W120 }
	binds adenine^c^ (1–2 residues)	Y333?	Y41?	∼380–384 (Q382, N384}	∼748–752; (E750}	∼410–412	∼802–806	∼135–139 (E137}
Region 2 STS	unclear	F336	F77	Y385	F753	F413	Y807	F140
region 2 STS	positions last E of [QE]XE via H-bonds, stabilizes oxacarbenium ion	S338A: 5	S79	S387	S755	S415	S809	S142
region 2 STS	stiffens active site via H bonds to nearby β-sheet	T339A: 20	T80	T388	T756	T416	T810	T143
region 2 STS	orients ARTT loop and [QE] of [QE]-X-E	S340A: 25	T81	S389	S757	S417	S811	S144
PN loop (region E)	stacking interaction with nicotinamide; mobile	F349A: 0; F349Y: 20		Y398	F768	F426	F819	F153
PN loop (region E)	binds N-phosphate	R352A: 0		R402	∼769–773	R437	R829	R166
ARTT loop	Target recognition, may reorient on ligand binding; can bind last E of [QE]XE	Y375	H125	Y426	Y790			Y186
ARTT loop	reaction step 3, substrate specificity; (mobile)	E378A: 0; E378D: 0; E378Q: 0	E128	Q429	E793	E461	Q884	E189
region 3 EXE	binds N-ribose, reaction step 1	E380A: 0; E380D: 0	E130	E431	E795	E463	E886	E191
Region 3	Unclear	L382	L59	L433	L797	L465	L888	L193

a These data apply to the homolog iota toxin and is rounded and reproduced from [33], [122].

b Possible range given, followed by residues most likely involved; protein backbone H-bonding.

c Possible range given, followed by residues most likely involved.

**Table 4:** Toxin regions that interact with target(s).

Name	region B active site loop	PN loop (or α3 helix)	ARTT loop
Chelt	N/A	82–99	104–129
Certhrax	295–314	390–402	420–430
Mav toxin	701–705	758–768	784–794
EFV toxin	361–370	418–436	452–462
TccC5	748–751	812–828	861–885
Vis toxin	82–91	145–164	180–190

**Figure 8:**
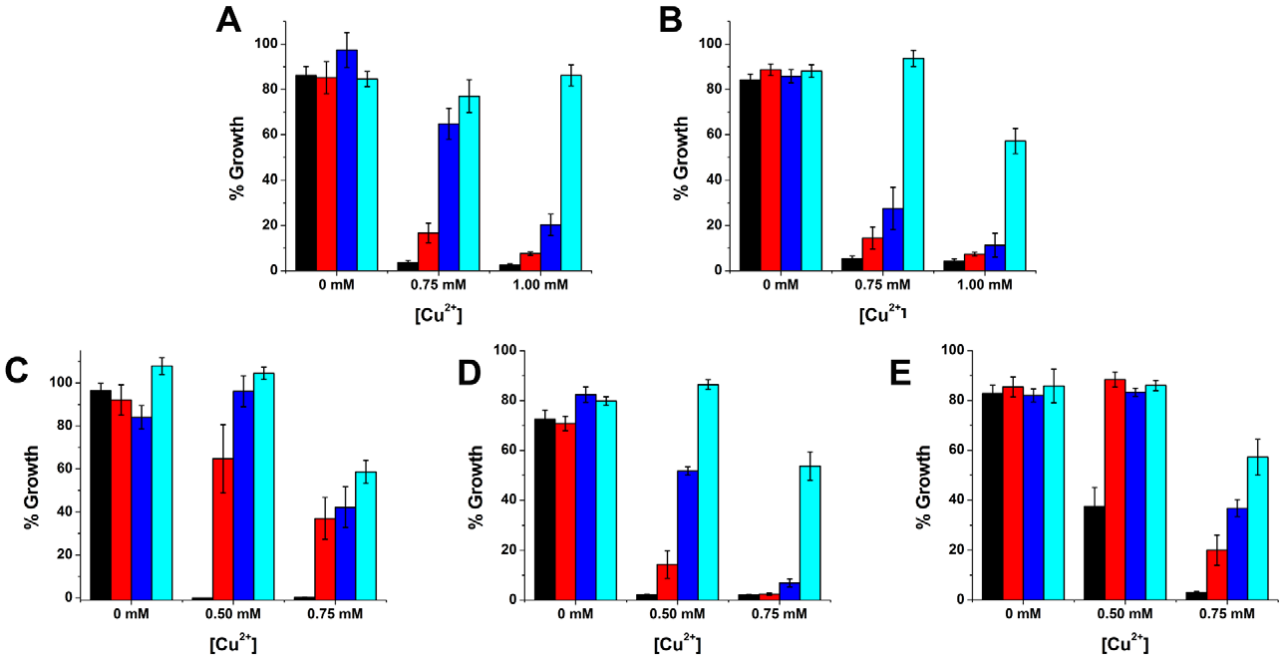
Growth-defective phenotype of yeast expressing the new ADP-ribosyltransferases. Growth of *S. cerevisiae* expressing WT or mutant toxin with substitutions to catalytic residues. The *CUP1* copper-inducible promoter drove toxin expression. (A) Catalytic domain of Chelt WT (black), E128A (red), E130A (dark blue) and E128A/E130A (light blue). (B) Certhrax WT (black), Q429A (red), E431A (dark blue) and Q429A/E431A (light blue). (C) EFV toxin WT (black), E461A (red), E463A (dark blue) and E461A/E463A (light blue). (D) TccC5 WT (black), Q884A (red), E886A (dark blue) and Q884A/E886A (light blue). (E) Vis toxin WT (black), E189A (red), E191A (dark blue) and E189A/E191A (light blue). Error bars show the SD of eight repeats.

Cell-based toxin expression in yeast, driven by the copper-inducible CUP1 promoter, shows cell death in the presence of the wild-type toxin. We observed mild growth restoration with the E128A mutant, dramatic growth restoration with the E130A mutant and near-complete growth restoration with the E128A/E130A double mutant (Figure 8A). The wildtype growth-defective phenotype clearly shows Chelt toxicity. Substitutions to E128 and E130 confirm that this toxicity is because of Chelt ADP-ribosyltransferase activity. Researches may modify Chelt in the future with the E128A and E130A substitutions – or produce recombinant forms including domain II only – to make vaccines similar to the commercial Dukoral [60].

### *B. cereus* Certhrax: Anthrax toxin with a different cell-killing strategy

Certhrax (UniProt Q4MV79) is encoded in *B. cereus* G9241. (A slightly larger relative exists in another *B. cereus* strain.) Most *B. cereus* strains are harmless or cause foodborne illness, but researchers have implicated this strain in several severe pneumonia cases [61]–[63]. Certhrax, a 476-residue, 55 kDa protein, is the first anthrax-related ADPRT toxin to our knowledge. It is 31% identical to lethal factor from *Bacillus anthracis*. The closest fold recognition match is to anthrax toxin lethal factor (LF, PDB 1J7N; J_3D-jury_ = 239, a high score reflecting a two-domain match). So we modeled Certhrax against LF. Certhrax has two domains: domain I (∼1–241) presumed to bind PA and domain II (∼242–476) is the toxin domain (Figures 4B and 5B).

*B. cereus* cells secrete this protein non-classically. Certhrax likely behaves similarly to LF in cell entry because of similarities in domain I, which is likely responsible for PA-binding. We describe a supposed model of Certhrax here using LF as a template [64]. Under harsh conditions, *B. cereus* forms spores that humans inhale into lung alveoli. Spores that escape from macrophages enter the lymph system where *B. cereus* germinates. Here, *B. cereus* produces protective antigen (PA, UniProt Q4MV80) that may bind Certhrax and edema factor (UniProt Q4MKW0). Both Certhrax and LF have a PA binding domain; sequence identity over this domain is 36%, within the safe zone of homology. But, Certhrax lacks the catalytic zinc metalloprotease domain of LF that proteolyzes mitogen activated protein kinase kinase (MAPKK or MEK). It contains a functional ADPRT domain instead of the vestigial ADPRT domain of LF (Figure S5 in Text S1). PA likely binds to ANTXR1/2 or LRP6 receptor. Furin proteolyzes PA so a PA heptamer can form. Certhrax and edema factor bind the PA heptamer and are translocated into the cell in a clathrin-coated pit. Low pH in the endosome causes a pore to form through which Certhrax and EF travel and enter the cytosol [64].

Domain II matches to iota toxin (PDB 1GIQ, J_3D-jury_ = 143). Fold recognition and phylogenetic analysis suggest similarities to C3-like toxins. We propose a likely mode of NAD^+^ binding, target binding and ADP-ribosylation based on alignment data and our modeling experiments (Figure 6B, Figure S4 in Text S1, Table 3). These H-bonds are likely: Q382 and N384 may bind to adenine, S344 binds to A-ribose, N288 and R292 bind to A-phosphate, R341 binds to A- or N-phosphate, T280 and E431 bind to N-ribose and R342 binds to nicotinamide. Active site residue Y398 in the flexible PN loop locks nicotinamide in the enzyme cleft during the reaction.

Certhrax likely recognizes its target through the region B active site loop (∼295–314), the PN loop (∼390–402) and the ARTT loop (∼420–430) (Table 4). The ARTT loop might plastically rearrange between the in and out conformation during target recognition. The second part may hinge on anchor residues S424 and Q429 to reposition Y426 to interact with the target substrate. We propose the reaction follows an S_N_1 alleviated-strain mechanism (Figure 7). First, E431 H-bonds to the N-ribose while phosphate electrostatic interactions hold the NAD^+^ in a conformation that favors oxacarbenium ion formation. Then Y284 induces a rotation about O_5D_-P_N_ bond of the oxacarbenium ion that reduces the nucleophile-electrophile distance. Finally, a target Glu or Asp stabilizes the N-ribose, Q429 stabilizes the target Asn or Gln and the target Asn or Gln attacks the oxacarbenium ion. Several residues hold the active site in place including: S387, which H-bonds to E431; T388, which stiffens the active site through H-bonding to a nearby β-sheet and S389, which orients the ARTT loop and Q429. Another conserved residue is Y279, which may participate in the reaction.

Toxin gene expression in yeast, driven by the CUP1 promoter, shows cell death in the presence of the wild-type toxin. We observed mild growth restoration with the Q429A and E431A mutants and near-complete growth restoration with the Q429A/E431A double mutant (Figure 8B). The wildtype growth-defective phenotype clearly suggests Certhrax toxicity. Substitutions to Q429 and E431 confirm that this toxicity is because of Certhrax ADP-ribosyltransferase activity. Researchers may eventually modify Certhrax with the Q429A and E431A substitutions – or produce recombinant forms of the toxin that include only the PA-binding domain I – to create vaccines similar to Biothrax that protects against *B. antracis* effects [65].

### *M. avium* Mav toxin: A possible type-VII secreted toxin may matter to AIDS patients

Mav toxin (UniProt A0QLI5) from *M. avium* strain 104 is a predicted ADPRT with possible relevance to AIDS patients who face a high risk of *M. avium* infections [66]. (Slightly larger relatives exist in *M. avium* subsp. paratuberculosis and *M. avium* subsp. avium ATCC 25291.) *M. avium* is both an environmental microbe and opportunistic pathogen causing chronic, pulmonary infections in immune-compromised individuals. Mav toxin is an 825-residue, 83 kDa protein with four putative domains: an ESAT6-like domain I (∼1–96), a predicted helical pore-forming domain II (∼97–439), a largely disordered domain III (∼440–674) and the toxin domain IV (∼675–825) (Figures 4C and 5C).

Domain I suggests secretion through the ESX (type VII) secretion system. This matches the non-classical secretion result. Fold recognition matches residues 1–95 to the 6 kDa early secreted antigenic target (ESAT-6; PDB 1WA8; J_3d-jury_ = 65; 16% identity). Virulent mycobacteria need the ESX secretion system for pathogenesis: ESX-1 deletion weakens virulence in *M. tuberculosis*, *M. bovis* and *M. marinum* [67]. ESAT-6 forms a heterodimer with the 10 kDa culture filtrate protein (CFP-10). Researchers believe the tight dimer binds an Rv3871-like ATPase for transfer to the Rv3877-like transmembrane pore through an Rv3870-like protein [68].

Domain II is α-helical, especially from 134–348. It might be a multi-helical bundle of short and long helices poised to form pores for target cell entry. Fold recognition matches are to the soluble domain of bacterial chemoreceptors (PDB 3G67, J_3d-jury_ = 93), a tropomyosin leucine zipper (PDB 2EFR, J_3d-jury_ = 78) and spectrin-like repeats (PDB 1QUU, J_3d-jury_ = 76). Domain III has slight propensity for forming β-sheets; but it is disordered. Its role is unknown, but it might recognize and bind cell-surface receptors. Combining domains II and III we found matches to the Cry insecticidal α-pore-forming toxins (a hard target for LOMETS, which provides a high-confidence match to PDB 1CIY).

Domain IV is the catalytic domain. Fold recognition suggests matches to Art2.2 (PDB 1GXY, J_3d-jury_ = 126). Mav, compared with iota toxin, has an 18-residue deletion after region 1 between P735 and A736. Also, and possibly affecting targeting, it has a two-residue PN-loop insertion (S765–S766).

We propose a likely mode of NAD^+^ binding, target binding and ADP-ribosylation based on alignment data and our modeling experiments. NAD^+^ binding (Figure 6C, Figure S4 in Text S1, Table 3) likely involves these H-bonds: E750 binds to adenine, N733 and possibly T732 bind to A-ribose, N695 and R699 bind to A-phosphate, R730 binds to A- or N-phosphate, T687 and E795 bind to N-ribose and G731 binds to nicotinamide. Active site residue F768 on the flexible PN loop locks the nicotinamide in the enzyme cleft during the reaction. Mav toxin recognizes its target using the region B active site loop (∼701–705), the PN loop (∼758–771) and the ARTT loop (∼784–794) (Table 4). The ARTT loop might plastically rearrange between the in and out conformation during this process. The first part of the ARTT loop, anchored between V784 and V787, is likely less flexible than the second part. The second part hinges on S788 and E793 to reposition Y790 to interact with the target substrate. We propose the reaction follows an S_N_1 alleviated-strain mechanism (Figure 7). First, E795 H-bonds to the N-ribose while phosphate electrostatic interactions hold the NAD^+^ in a conformation that favors oxacarbenium ion formation. Then Y691 induces a rotation about O_5D_-P_N_ bond of NAD that reduces the nucleophile-electrophile distance. Finally, a target Glu or Asp stabilized the N-ribose, E793 stabilizes the target Arg and the target Arg attacks the oxacarbenium ion. Several residues hold the active site in place including: S755, which H-bonds to E795; T756, which stiffens the active site through H-bonding to a nearby β-sheet and S757, which orients the ARTT loop and E793. Also, Y686 stabilizes E795.

Neighbourhood and co-occurrence evidence suggest Mav may interact with the exported repetitive protein (UniProt A0Q9B3) – suggested as a virulence factor in *Mycobacteria* [69] – and several putative uncharacterized proteins. Cloning problems frustrated cell-based characterization in yeast. As well, we have several concerns about this prediction: a characteristic WXG motif is lacking in domain I and the whole protein is unusually long for ESX-1 secretion. Perhaps Mav toxin uses a variant of the ESX-1 system (ESX-2 to ESX-5). Also, the genetic context suggests a haloacid dehalogenase-like hydrolase is encoded nearby, making de-ribosylation reactions a concern. But, we believe this putative toxin is worth presenting despite these issues because of its potential health implications.

### *E. faecalis* EFV toxin: A new toxin from a superbug

EFV toxin (UniProt Q838U8) is a medically relevant ADPRT candidate from a vancomycin-resistant *E. faecalis* strain, V583 [70]. This strain produces cytolysin toxin [71] and causes urinary infection, bacteremia and endocarditis [72]. A slightly smaller relative exists in *Enterococcus faecalis* CH188. EFV toxin itself is a 487-residue, 56 kDa protein and has a needle-like helical domain I (∼1–309) and catalytic domain II (∼310–487) (Figures 4D and 5D).

The toxin is non-classically secreted (i.e., without a signal peptide). A type IV secretion system has been identified in *E. faecalis* [73], but it is unclear if it mediates EFV toxin secretion. Genetic context suggests that EFV toxin may more likely travel through a phage infection conduit to target cells. Neighbourhood, gene fusion and co-occurrence evidence suggest it may interact with portal proteins (UniProt Q838U9 and Q833E4), a scaffold protein (Q838U5), a major tail protein (Q835T7), a Cro/CI family transcriptional regulator (Q835K8) and several putative uncharacterized proteins. The phage origin makes it unclear whether EFV toxin acts mainly against bacterial or eukaryotic targets.

Domain I bears large sequence similarity to phage minor head region from 147–268 that suggests a possible phage origin. The phage head match is reminiscent of the dual role of Alt in bacteriophage T4 as both a phage head structure component and a RNA-polymerase targeting ADPRT [74]. Fold recognition on domain I suggests matches to spectrin (PDB 1U4Q, J_3d-jury_ = 49; a hard target for LOMETS, which provides this high-confidence match) and weaker matches to the pore-forming domain of colicin s4 (PDB 3FEW, J_3d-jury_ = 42). Also genetic context suggests similarities to the bacteriophage P22 needle implicated in cell-envelope penetration [75].

Domain II is 25% identical to *Bacillus thuringiensis* VIP2 over 166 residues. EFV toxin has C2-like character based on its phylogenetic branching. It also has a region 3 EXE sequence pattern that suggests an Arg target. Fold recognition suggests that its closest structure match is to C2-I (PDB 2J3Z, J_3D-jury_ = 158).

The efforts of the Midwest Center for Structural Genomics have failed to produce a structure. We propose a likely mode of NAD^+^ binding, target binding and ADP-ribosylation based on alignment data and our modeling experiments (Figure 6D, Figure S4 in Text S1, Table 3). These H-bonds are likely: S397, N399 or E400 binds to A-ribose, N354 and R358 bind to A-phosphate, R394 binds to A- or N-phosphate, T346 and E463 bind to N-ribose and G395 binds to nicotinamide. Active site residue F426 in the PN loop locks the nicotinamide in the enzyme cleft during the reaction. EFV toxin recognizes its target using the region B active site loop (∼361–370), the PN loop (∼418–436) and the ARTT loop (∼452–462) (Table 4). The ARTT loop might plastically rearrange between the in and out conformation during this process, hinging on S456 and E461. Compared with iota toxin, and possibly influencing target recognition, EFV toxin has a 22-residue deletion in region F (between regions 1 and 2) between A403 and I404. Also possibly influencing targeting, EFV toxin has a six-residue PN loop insertion (E424–F429). We propose the reaction follows an S_N_1 alleviated-strain mechanism (Figure 7). First, E463 H-bonds to the N-ribose while phosphate electrostatic interactions hold the NAD^+^ in a conformation that favors oxacarbenium ion formation. Then F350 likely induces a rotation about the O_5D_-P_N_ bond of the oxacarbenium ion bond to reduce the nucleophile-electrophile distance. Finally, a target Glu or Asp stabilizes the N-ribose, E461 stabilizes the target Arg which attacks the oxacarbenium ion. Several residues hold the active site in place including: S415 which H-bonds to E463; T416, which stiffens the active site through H-bonds to a nearby β-sheet and S417, which orients the ARTT loop and E461. Also, Y345 stabilizes E463. Other potential active site residues include T346, E412 and F426.

EFV toxin expression in yeast, driven by the CUP1 promoter, shows cell death in the presence of the wild-type toxin. We observed dramatic restoration growth with the E461A and E463A mutants and near-complete growth restoration with the E461A/E463A double mutant (Figure 8C). The wildtype growth-defective phenotype clearly shows EFV toxin toxicity. Substitutions to E461 and E463 confirm that this toxicity is because of EFV toxin ADP-ribosyltransferase activity.

### *P. luminescens* TccC5: An ADPRT associated with a toxin complex

TccC5 (UniProt Q7N7Y7) is an ADPRT from *P. luminescens* TT01 that we previously suggested as an ADPRT toxin [4], which has gained significant attention recently [53]. Is distinct from the recently reported Photox [56], but a close relative also exists in the W14 strain.

TccC5 is 938-residue, 105 kDa protein and has four domains: domain I (∼1–341), domain II (∼342–675), domain III (∼676–738) and domain IV (∼739–938) (Figures 4E and 5E). This toxin is non-classically secreted. Fold-recognition matches to TccC5 are to various tandem seven-bladed β-propellers, including the actin-interacting protein (PDB 1NR0; J_3D-jury_ = 71) and the Sro7 exocytosis regulator (PDB 2OAJ, a high-confidence LOMETS match). These proteins are WD40 repeat-containing proteins (SCOP b.69.4, CATH 2.130.10.10). Also, we found matches to several tandem seven-bladed β-propeller xyloglucanase structures (PDB IDs 3A0F, 2EBS, 2CN2; SCOP b.69.13; CATH 2.130.10.140) that hydrolyze polysaccharides.

Fold recognition on domain I, a hard target, produces matches to various β-propellers such as βγ-dimer of the heterotrimeric G-protein transducin (PDB 1TBG, LOMETS high-confidence match), oxidoreductases (PDB 1FWX, J_3d-jury_ = 123), outer surface protein OspA (PDB 1FJ1, J_3d-jury_ = 83, LOMETS high-confidence match to 2FJK), Tyr-Val-Thr-Asn (YVTN) domain from an archaeal surface layer protein (PDB 1L0Q, a high-confidence LOMETS match), lyases (e.g., streptogramin B lyase, PDB 2QC5, a LOMETS high-confidence match; and virginiamycin B lyase, PDB 2Z2P, J_3d-jury_ = 51), among others. Function prediction suggests domain I contains two YD repeats possibly involved in binding carbohydrates and heparin. Also, domain I contains a lipocalin pattern, hinting at a connection to small-molecule transporters.

Fold recognition on domain II, also a hard target, shows there may be a second β-propeller after the first. Matches are to various β-propellers including OspA, YVTN from an archaeal surface layer protein and the extracellular domain of LDL receptor (PDB 1N7D, a high-confidence LOMETS match), among others. The C-terminal end of domain II appears to have recombination hot spot (Rhs) repeats employed in other secreted bacterial insecticidal toxins and eukaryotic intercellular signalling proteins, and often involved in ligand binding. Rhs suggests horizontal transfer; it is related to YD repeats and also often contains VgrG, a type VI secretion protein. β-propellers are structurally conserved but functionally diverse, so it is difficult to pinpoint exact functions for domains I and II. While the exact role of these domains is unclear, a likely role is gaining cell entry. Domain III seems helical with unknown function.

TccC5 domain IV best matches SpvB but identity is only 25% over the toxin core, making TccC5 among the most novel toxins discussed here. Fold recognition results suggest that TccC5 is similar to C3bot2 (PDB 1R45, J_3d-jury_ = 92) throughout the catalytic domain. Recently, Lang *et al.* identified the cellular target as RhoA Q63 [53].

We propose a likely mode of NAD^+^ binding, target binding and ADP-ribosylation based on alignment data and our modeling experiments. TccC5 binds NAD^+^ through hydrogen bonds, hydrophobic interactions and aromatic interactions (Figure 6E, Figure S4 in Text S1, Table 3). We propose these H-bonds: T777 binds to A-ribose, N742 and R746 bind to A-phosphate, R774 binds to A- or N-phosphate, R829 may bind N-phosphate, T735 and E886 bind to N-ribose and V775 binds to nicotinamide. Active site residue F819 in the flexible PN loop locks the nicotinamide in the enzyme cleft during the reaction. TccC5 recognizes RhoA using the region B active site loop (∼748–751), the PN loop (∼812–828) and the ARTT loop (∼861–885) (Table 4). The ARTT loop might plastically rearrange between the in and out conformation during this process. Compared to SpvB, TccC5 has several key differences that may influence targeting including: a 30 amino acid deletion in region B between I750 and T751, an eight-residue insertion in the PN loop (F819–S826) and a 32-residue insertion in the ARTT loop between A854 and E885. Other variations include a five-residue insertion between I779 and K783 and two deletions that follow the ARTT loop, namely, three residues between R901 and H902 and two residues between I914 and K915. We propose the reaction follows an S_N_1 alleviated-strain mechanism (Figure 7). First, E886 H-bonds to the N-ribose while phosphate electrostatic interactions hold the NAD^+^ in a conformation that favors oxacarbenium ion formation. The reaction's progress is unclear. S738 might induce a rotation about the O_5D_-P_N_ bond of the oxacarbenium ion to reduce the nucleophile-electrophile distance. A RhoA Glu or Asp likely stabilizes N-ribose, TccC5 Q884 likely stabilizes RhoA Asp, and finally RhoA Q63 attacks the oxacarbenium ion. Several residues hold the active site in place including: S809, which H-bonds to E886; T810, which stiffens the active site through H-bonding to a nearby β-sheet and S811, which orients the ARTT loop and Q884. Also, Y734 stabilizes E886.

Co-occurrence, neighbourhood, gene fusion and recent evidence [53], suggest that TccC5 exists as part of a toxin complex with the TcdA1 toxin and TcdB2 potentiator. Full activity depends on these partners [76].

TccC5 expression in yeast, driven by the CUP1 promoter, shows cell death in the presence of the wild-type toxin. We observed mild growth restoration with the Q884A mutant, dramatic growth restoration with the E886A mutant and near-complete growth restoration with the Q884A/E886A double mutant (Figure 8D). The wildtype growth-defective phenotype clearly shows TccC5 toxicity. Substitutions to Q884 and E886 confirm that this toxicity is because of TccC5 ADP-ribosyltransferase activity.

### *V. splendidus* Vis: A minimal ADPRT toxin

Vis (UniProt A3UNN4) is an ADPRT from a known pathogen, *V. splendidus* 12B01, which causes vibriosis and afflicts oysters. Similar proteins exist in *Vibrio harveyi* strains HY01 and BB120, *Photobacterium* sp SKA34 and *Photobacterium angustum* S14. Vis toxin is 30% identical to VopT from *Vibrio parahaemolyticus*. This single-domain toxin has 249 residues and is 28 kDa. It harbors a secretion signal peptide with a cleavage site between position 18 and 19 (Figures 4F and 5F). Fold recognition matches it to iota toxin (PDB 1GIQ, J_3D-jury_ = 135). Vis entry into target cells is unclear. It may travel through a transporter, be aided by other pore-forming toxins or be directly released into the cytosol after *V. splendidus* invasion.

We propose a likely mode of NAD^+^ binding, target binding and ADP-ribosylation based on alignment data and our modeling experiments. NAD^+^ binding (Figure 6F, Figure S4 in Text S1, Table 3) likely involves these H-bonds: E137 binds to adenine, W120 may bind to A-ribose, N76 and R80 bind to A-phosphate, R117 binds to A- or N-phosphate, S68 and E191 bind to N-ribose and G118 binds to nicotinamide. Active site residue F153 in the flexible PN loop locks the nicotinamide in the enzyme cleft during the reaction. Vis recognizes its target using the region B active site loop (∼82–91), the PN loop (∼145–164) and the ARTT loop (∼180–190) (Table 4). Vis has a 24-residue deletion after the region 1 Arg between K122 and L123. Also, and possibly affecting targeting, it has a four-residue region B insertion between V89-A92 and an eight-residue insertion in the PN loop between E148 and V155. The ARTT loop might plastically rearrange between the in and out conformation during target recognition. The first part of the ARTT loop is anchored between hydrophobic residues I180 and L183 and is likely less flexible than the second part. This second part, which hinges on S184 and E189, likely repositions Y186 to interact with the target substrate. We propose the reaction follows an S_N_1 alleviated-strain mechanism (Figure 7). First, E191 H-bonds to the N-ribose while phosphate electrostatic interactions hold the NAD^+^ in a conformation that favors oxacarbenium ion formation. Then Y72 induces a rotation about O_5D_-P_N_ bond of the oxacarbenium ion that reduces the nucleophile-electrophile distance. Finally, a target Glu or Asp stabilizes the N-ribose, E189 stabilizes the target Arg or Cys which attacks the oxacarbenium ion. Several residues hold the active site in place including: S142, which H-bonds to E191; T143, which stiffens the active site through H-bonds to a nearby β-sheet and S144, which orients the ARTT loop and E189. Also, Y76 stabilizes E188. F153 promotes NAD^+^ binding and glycohydrolase activity. F67 is another conserved residue possibly involved in the reaction.

Vis toxin expression in yeast, driven by the CUP1 promoter, shows cell death in the presence of the wild-type toxin. We observed mild growth restoration with the E189A and E191A mutants and dramatic growth restoration with the E189A/E191A double mutant (Figure 8E). The wildtype growth-defective phenotype clearly suggests Vis toxicity. Substitutions to E189 and E191 confirm that this toxicity is because of Vis toxin ADP-ribosyltransferase activity.

### Conclusion

We have combined computer results with cell-based data to improve toxin discovery and characterization. The six new toxins described here are a significant addition to the list of known ADPRTs. Interested readers may refer to Text S1 for further discussion of trends in structure and function.

Future toxin discoveries will involve not only new entries to public sequence and structure databases, but also updates to the search pattern and perhaps even new folds. For example, Johnson *et al.* recently showed the region 2 STS motif is not strictly needed in an *M. penetrans* ADPRT [55]. Also, the PARP10 ADPRT does not need the hallmark “catalytic Glu” because it uses a substrate-assisted mechanism [77]. AexU from *Aeromonas hydrophila* [78], [79] may reveal a new ADP-ribosylation fold: our preliminary fold-recognition tests suggest it does not adopt the typical ADPRT fold.

We must do much work to characterize the new toxins *in vitro*. One challenge is developing a way to reliably overcome expression, purification and solubility problems, which seem typical in this family. If we can overcome these problems, we may pinpoint structure details through X-ray crystallography in cases where the toxin is amenable such techniques. Finding intracellular targets will also aid in elucidating functional details. Time-resolved crystallography, NMR spectroscopy and QM/MM simulations may one day further reveal reaction dynamics [80]. Our efforts in cell-based characterization may involve more complete *in vivo* characterization where we give purified toxin to suitable target cells or model organisms. Applying knowledge of these new toxins to improve human health and agricultural production is a large-scale but worthwhile challenge.

## Methods

### Data mining: Searching for new ADPRT toxins

We used remote homolog detection strategies to find ADPRTs within the set of all known sequences. Authors have reviewed [81], [82] and benchmarked [83] these strategies. Often the only way to find remote homologs to a query sequence is through structure links, so structure prediction and remote homolog detection often rely on the same strategies. One effective strategy is to pair structure prediction with matches to consensus patterns.

Russell *et al.* described the leading structure classification databases [84]. We used the Structural Classification of Proteins (SCOP) [85] and Class Architecture Topology Homology (CATH) [86] databases. We extracted structure codes for the ADPRT family from these databases for further searches. We used these SCOP codes: d.166.1.1 (mART), d.166.1.2 (PARPs), d.166.1.3 (ARTs), d.166.1.4 (AvrPphF ORF2, a type III effector), d.166.1.5 (Tpt1/KptA), d.166.1.6 (BC2332-like) and d.166.1.7 (CC0527-like). We used these CATH codes: 3.90.175.10 (DT Group mART), 3.90.176.10 (C2- and C3-like mARTs, ARTs), 3.90.210.10 (CT-PT-like mARTs) and 3.90.182.10 (Anthrax_PA-like). Teichmann *et al.* described several fold-recognition databases [87]. To get a putative ADPRT toxin list, we searched the structure classification codes for known ADPRTs against such databases, including Gene3D [50] and SUPERFAMILY [51].

### Data mining: Filtering hits

We filtered the resulting sequences for ADPRT toxins by keeping only bacterial hits using NCBI taxon IDs, keeping only hits from pathogens using gene ontology data and the GOLD database [1], keeping only hits that tested positive for secretion using SignalP 3.0 or Secretome P 2.0 and keeping only hits that had the conserved ADPRT pattern using ScanProsite [88] with this regular expression: [YFL]-R-X(27,60)-[YF]-X-S-T-[SQT]-X(32,78)-[QE]-X-E. We formed this pattern strictly using known 3D structures in 3dLOGO and changing the resulting regular expression to ensure that it captured known ADPRT toxins in ScanProsite searches. We kept only hits with less than 50% identity to a known toxin and further reduced the list by clustering at the 50% identity level. We checked genetic context for hydrolases using Entrez Gene [89] and removed sequences where one was encoded nearby. (Ribosylhydrolases and ribosylglycohydrolases can de-ribosylate proteins. Hydrolases may suggest a regulatory cycle or toxin-antitoxin selfish genetic entities [90].) We selected several interesting examples to characterize and discuss. We ranked the final toxin list in order of decreasing ISI Web of Knowledge hits to the species name.

### Multiple sequence alignment and phylogenetic analysis

For both the C2-C3-like toxins and the CT-PT-like toxins, we aligned known and new toxins using 3D-Coffee [91], we visualized the alignment using ESPript [92], we curated it to remove positions with gaps using Phylogeny.fr [93] and converted it to LOGO format using WebLOGO [94]. We produced a percent identity matrix using ClustalX [95] to reveal the relationships between the new and known ADPRT toxins.

We curated an alignment containing all ADPRT toxins by removing positions with gaps to prepare it for phylogenetic analysis by Bayesian inference with the MrBayes algorithm [96]. The likelihood model included six substitution types with invariable and gamma rate variation across sites. Markov chain Monte Carlo parameters included 10,000 generations, sampling a tree every 10 generations. We discarded the first 250 trees sampled.

### Structure prediction: Fold recognition

Fisher reviewed fold recognition servers [97]. We sent the putative ADPRT toxins to fold-recognition meta-servers including: 3D-jury [98], Pcons [99], Genesilico [100], LOMETS [101] and Atome2 [102]. Sequences with top hits to ADPRT toxins or ADPRT-related structures (e.g. ART, PARP, LF, etc.) remained on the list. We recorded the J_3D-jury_ and structure match for each sequence. J_3D-jury_> = 40 is usually correct, but ideally we like it to be 100 or more for strong structure matches. We reassessed sequences showing no match to ADPRT-like proteins by using sliding-window fold–recognition (see structure prediction: domain organization below). If no match to an ADPRT-related structure appeared, we removed them from the list. We checked ScanProsite matches against fold-recognition results, and adjusted them to ensure that we correctly identified the region 1 Arg, region 2 “STS” motif and region 3 ARTT motif.

### Structure prediction: Domain organization

The CASP7 competition compared domain prediction tools [103]. We present domain assignments and boundaries that often differ from data in public domain databases or are unavailable. We used top performer DOMAC (Accurate, Hybrid Protein Domain Prediction Server). It uses both template-based and *ab initio* methods and uses a PSI-BLAST generated profile to find templates. For significant matches it uses MODELLER for modeling and the protein domain parser (PDP) for domain parsing. If it does not find matches, it relies on neural networks or support vector machines (SVMs) [104]. We manually adjusted these results to match the sliding-window fold recognition data, testing sliding windows of 50, 75, 100, 150, 200, 250, 300, 350 etc. amino acids on the fold-recognition meta servers to identify boundaries and fold type for the non-toxic domains. We mapped PDB hits to SCOP and CATH codes and interpreted the results to understand cell-entry strategies [105].

### Structure prediction: Comparative modeling

Nayeem *et al.* compared modeling software [106]. Prime works best for modeling in low sequence identity cases. But Modeller [107] is widely used, updated often and freely available, so we chose it for our work. For each candidate ADPRT, we used the alignments in Figures S1 and S2 in Text S1 and 3D-jury to select a suitable input alignment of the new toxin against a known template. We inspected the input alignments to ensure that we had properly aligned regions B, 1, 2 and 3.

We modeled NAD^+^-bound structures using MODELLER and alignments to an NAD^+^-bound template: C3bot1 (PDB 2A9K) [108], Iota toxin (PDB 1GIQ) [33], SpvB (PDB 2GWL) [109], EDIN-B (PDB 1OJZ) [110], CdtA (PDB 2WN7) [111], Art2.2 (PDB 1OG3) [112], Vip2 (PDB 1QS2) [39] and cholera toxin (PDB 2A5F) [113]. Except for Chelt, we used all templates to find invariant features between the resulting models and interpret the new toxins based on consistent NAD^+^-binding patterns.

We modeled full-length ADPRT structures using I-TASSER, the top-ranked program for fully-automated structure prediction in CASP7. It combines folds and supersecondary structures selected from the PDB with *ab initio* loop models. These elements are reassembled and refined to produce the final model. When I-TASSER failed to produce a result matching the sliding-window fold recognition data (four cases), we selected suitable templates from this fold recognition data. We docked the templates using HADDOCK [114] and used them as MODELLER input. Where appropriate, we used VTFM and MD to optimize the models and repeated the modeling cycle at least two times to achieve an adequate objective function (>1×10^6^). We refined loops automatically after model building and ranked them by Discrete Optimized Protein Energy (DOPE) statistical potentials to find the top model. We visualized the models using PyMol.

Laskowski *et al.* reviewed model quality assessment programs (MQAPs) [115]. We assessed the ADPRT models using MetaMQAPII, a meta-server that considers results from VERIFY3D, PROSA, BALA, ANOLEA, PROVE, TUNE, REFINER and PROQRES [116]. We also gathered model data using MolProbity [117].

### Function prediction: NAD^+^ binding

We assessed NAD^+^ binding using crystal structures solved with NAD^+^ in the active site: C3bot1 (PDB 2A9K) [108], Iota toxin (PDB 1GIQ) [33], SpvB (PDB 2GWL) [109], EDIN-B (PDB 1OJZ) [110], CdtA (PDB 2WN7) [111], Art2.2 (PDB 1OG3) [112], Vip2 (PDB 1QS2) [39] and cholera toxin (PDB 2A5F) [113]. We used LigPlot [118] on the PDBsum server [119]) to visualize the usual interactions in ADPRT NAD^+^ binding. We used the 3dLOGO [120] software to reveal equivalent positions in these structures. We used conserved residues from the alignment involved in typical NAD^+^ binding interactions in the known ADPRTs to identify the equivalent residues in the new ADPRTs. We also analyzed our NAD^+^-bound models and compared the ADPRTs modeled directly against the NAD^+^-bound templates using Modeller [107].

### Function prediction: Reaction mechanism

We developed the ADPRT toxin reaction mechanism for the new toxins using the S_N_1 alleviated-strain model, first proposed by Tsuge *et al.*, that many believe is widely relevant to the entire family [34]. As for NAD^+^ binding we used 3DLOGO [120] to reveal equivalent positions in these structures: C3bot1 (PDB 2A9K), Iota toxin (PDB 1GIQ), SpvB (PDB 2GWL), EDIN-B (PDB 1OJZ), Art2.2 (PDB 1OG3), Vip2 (PDB 1QS2) and cholera toxin (PDB 2A5F). We also matched residues involved in the iota toxin mechanism to residues in SpvB, EDIN-B and C3bot1 and to the new toxins using 3D-jury results. We exploited conservation of the hallmark catalytic Glu for step 1, a conserved aromatic (usually Tyr, but sometimes Phe) for step 2 and the secondary Glu or Gln for step 3. We also used the rule that region 3 [QE]XE pattern appears as EXE in ADPRTs that ribosylate Arg and as QXE in ADPRTs that ribosylate Asn, Gln or Cys.

### Cell-based validation

We cultured *Saccharomyces cerevisiae* strain W303 (*MATa*, *his3*, *ade2*, *leu2*, *trp1*, *ura3*, *can1*) on yeast-peptone-dextrose or synthetic dextrose (SD) drop-out medium. We performed the yeast growth-defective phenotypic test and quantified growth as previously described [48].

## Supporting Information

Text S1 Supplementary discussion, figures, tables and data.
(4.63 MB PDF)
