# Supplementary material for: Cholera- and Anthrax-Like Toxins Are among Several New ADP-Ribosyltransferases
Source: PLoS Comput Biol. 2010 Dec 9;6(12):e1001029. doi: 10.1371/journal.pcbi.1001029 (PMC3000352; doi:10.1371/journal.pcbi.1001029)
Supplement: Text S1 — Supplementary discussion, figures, tables and data. (4.63 MB PDF) [file pcbi.1001029.s001.pdf]

Text S1

Supplementary Discussion

Fold recognition, structure prediction and modeling

Locating the ADPRT pattern in a protein sequence reveals the catalytic domain. Pattern matches, adjusted to be consistent with sequence alignment, reveals specific catalytic residues.

Fold recognition is a key step toward understanding ADPRT structure and selecting modeling templates. The number of correctly predicted residues ($C_\alpha \leq 3.5$Å) correlates with $J_{3D\text{-}jury}$, according to the formula: $N = 0.86J_{3D\text{-}jury} + 9.91$ [S1]. A typical ADPRT domain, at 200 residues, would therefore receive a "perfect score" of about 220. In practice, we find predictions are strong for $J_{3D\text{-}jury}$ above 100 and often acceptable for $J_{3d\text{-}jury}$ >= 40. Fold recognition results depend on lengths and the part of a sequence tested. It is important to fragment the test sequences in various ways and run many jobs (Dataset S1). Also, we expect fold recognition to become more reliable over time as new non-ADPRT structures lessen wrong fold assignments.

We produced two model types: full-length and active site. We conservatively assume the new toxins, based on how we searched for them, follow existing sequence-structure-function trends especially for key residues. We show these trends – based on structure and biochemical investigations spanning more than a century [S2, S3] – in Figures 1 and 2. The full-length models (Figure 5) merge data from different templates. We do not intend to imply any specific domain orientation or any details on specific embellishments to the core fold. Active-site models (Figure 6) are our most accurate toxin models because structure conservation is highest in this region. They help in understanding likely NAD^+ binding modes and aid in envisioning the reaction mechanism.

Model accuracy depends on suitable template selection and alignment quality. High accuracy models may contain main chain distortions or shifts, incorrectly packed side chains and incorrectly modeled loops. Medium accuracy models may contain more alignment errors and errors in loop regions. We intend low-accuracy models as a general structure framework only. They are not suited for detailed analysis because they may have more significant errors [S4]. Estimates of the global distance test total score (GDT-TS) – which counts Cα pairs that have a distance <1, 2, 4, 8 Å after overlap – can be used to assess model quality. Larger values are better. Values >40 imply the correct fold and values >84 imply reliable use in molecular replacement [S5]. Our best models resulted from high sequence identity and high structure resolution. We list specific quality scores in Tables S2 and S3.

Toxin secretion, cell entry and domain organization

Domain boundary prediction is difficult [S2]. Domain prediction accuracy varies according to the method used. In DOMAC, domain number is 75% accurate for template-based cases and 46% accurate for *ab initio* cases. Domain boundaries, for template-based cases have a 50% specificity and 77% sensitivity. For *ab initio* cases, this drops to 27% and 14% respectively [S6]. We expect our sliding window fold recognition results to have much higher accuracy, perhaps even approaching new breakthrough methods [S7]. But clearly ADPRT toxin domain combinations are more wide-ranging than previously thought (Figure 4).

Domain organization may be driven by different secretion strategies. Clearly, the ADPRT toxins must leave the pathogen cell and enter the host cell to have an effect. Thus, we expect the pathogen cell to secrete the toxins. Often secretion involves a type II, III or VI secretion system, and toxin domain organization may reflect the secretion strategy. N-terminal disorder, for example, may suggest interaction with a secretion chaperone. In all cases, neural network evidence from SecretomeP suggests secretion, but the mechanism is not always clear. SignalP sometimes suggests a signal peptide (it has an accuracy of 0.95 for Gram negative organisms and 0.98 for Gram positive organisms). In other cases, we rely more on genetic context to learn about secretion.

Another explanation of the varying domain organization is different cell entry strategies. Small ADPRT toxins lacking receptor binding and membrane translocation machinery may be (i) associated with receptor-binding and membrane translocation domains that exist as separate polypeptides (ii) injected directly by a secretion system (iii) aided by other pore-forming toxins (iv) introduced through a transporter or (v) act at the cell-surface. Larger ADPRT toxins, however, may have directly attached receptor binding and membrane translocation domains that remain with the toxin domain or are cleaved off through proteolysis. They may also have an activation moiety needed for binding an activating protein. We speculate that alpha helical regions may have a greater tendency to be pore-formers, while beta sheet regions may suggest receptor binding. Predicting non-catalytic domains structures provides insight into the cell entry strategies of the toxins.

Toxin activation, NAD+ binding and the reaction mechanism

Often ADPRT toxins do not require activation, but sometimes they do. Common modes of ADPRT toxin activation include: (i) binding to factor activating ExoS (FAS) (for the ExoS-like subgroup), (ii) a combination of proteolysis, reduction and ADP-ribosylating factor (ARF) binding (for cholera toxin and heat labile toxin), (iii) a combination of ATP and reduction in pertussis toxin.

We conservatively assume that NAD^+ binding in the new ADPRTs will follow trends in previous structures. Specific, conserved residues bind to A-phosphate, N-ribose and nicotinamide. There is little variation except for adenine ring orientation, and no conserved residues exist for adenine and A-ribose binding. Predictions for the adenine ring are difficult but less important to ADPRT toxin function. Variable NAD^+ binding at the A-ribose may exist because H-bonding is to the protein backbone. Also, H-bonds to the A-ribose are within a loop region instead of a side-chain from a conserved

secondary structure element. H-bonding is sparse at the nicotinamide ring, suggesting aromatic or hydrophobic interactions position this ring.

The reaction mechanism follows three steps: nicotinamide proteolysis to form the oxacarbenium ion, rotation about the O_{5D}-P_N bond to relieve strain in the bound NAD^+ substrate which forms another ionic intermediate and, finally, nucleophilic attack of the second intermediate by the target. Based on the iota toxin alleviated-strain model, a Tyr in the second step induces a rotation of the oxacarbenium ion about the phosphate single bond (O_{5D}-P_N) and helps reduce the nucleophile-electrophile distance. Interestingly, this Tyr is lacking in EFV toxin; F350 replaces it. Previous work has shown activity is retained on mutating Tyr to Phe in Iota toxin [S8]. T71 replaces the Tyr in Chelt and S738 replaces the Tyr in TccC5, suggesting that substitution to polar residues may not reduce activity as much as expected.

Challenges for *in vitro* characterization

The calculated solubility probability ranges from 0.06 to 0.611. This tendency toward insolubility matches our experiences at the lab bench. Disordered regions outside the catalytic domain may obstruct solubility and crystallization efforts.

The macromolecule target for ADPRT toxin ADP-ribosylation is relevant for understanding ADPRT toxicology and for lab investigations. Such knowledge is academically important, but we do not need it to develop vaccines or inhibitors. There are many ADP-ribosylation superfamily targets. They include DNA binding proteins, membrane proteins, extracellular body fluid proteins, RNA polymerase, dinitrogenase reductase, DNA and rifamycin. ADPRT targets are wide-ranging, including many G-proteins (eEF2, Ras, Rho and Gs-α, etc.), actin and RNA-binding proteins. Pinpointing the possible targets is challenging and in its infancy from a predictive standpoint.

Target prediction accuracy currently depends on sequence identity. We can sometimes assign target substrate based on several facts. First, target specificity seems partly or fully contained in the region B loop, PN loop and the ARTT loop (Table 4) [S9]. Second, the region 3 EXE toxins target specific Arg residues on targets; QXE toxins target Rho or specific target Asn, Gln or Cys residue [S9]. Third, the E.C. number of an enzyme rarely changes above 40% sequence identity [S10].

One trend is that many ADPRT targets are in the G-protein clan (Pfam CL0017) [S11, S12]. Specific families targeted in the past include the elongation factor Tu GTP binding domain family (Pfam GTP_EFTU, PF00009): DT-like toxins target eEF2 and MTX targets EF-TU; the Ras family (Pfam PF00071): ExoS-like toxins target Ras and C3-like toxins target Rho; and the Gα family (Pfam PF00503): CT-like toxins target Gαs and PT-like toxins target Gαi. The G-protein clan has many other families that may be targets according to this trend. These include HSR1-related GTP-binding proteins (Pfam MMR_HSR1, PF01926); the dynamin family involved in endocytosis (Pfam Dynamin_N, PF00350); the septin family involved in cytokinesis (Pfam PF00735); the Miro family involved in mitochondrial motility (Pfam PF08477); the ATP binding proteins (Pfam ATP_bind_1, PF03029); the bacterial resistance proteins (Pfam AIG1, PF04548), the nucleolar GTP-binding proteins (Pfam NOG1, PF06858); interferon-inducible GTPases (Pfam IIGP, PF05049); the signal recognition particle receptor beta subunit (Pfam SRPRB, PF09439); and the DUF258 family (Pfam PF03193). Interestingly, the family of ADP-ribosylation factors (Pfam Arf family; PF00025) is also in the G-protein clan.

Another common target is the actin family (Pfam PF00022 in Pfam clan CL0108, Actin_ATPase) [S11, S12]. Some actin-targeting toxins ADP-ribosylate all actin isoforms, including α-actin while others ADP-ribosylate β/γ-non-muscle G-actin and γ-smooth muscle actin (e.g. C2 toxin) [S13]. GRP7, targeted by HopU1, is part of the RNA-recognition motif family (Pfam RRM_1, PF00076) within the RRM clan (Pfam CL0221) [S12, S14]. Other ADPRTs have different targets: PARPs target DNA binding proteins; ARTs target membrane proteins and proteins of extracellular body fluid; Alt, ModA and ModB target RNA polymerase; DRATs target dinitrogenase reductase; pierisin targets DNA and Arr targets rifamycin [S15].

References

S1. Kajan L, Rychlewski L. (2007) Evaluation of 3D-jury on CASP7 models. BMC Bioinformatics 8: 304.

S2. Tress M, Cheng J, Baldi P, Joo K, Lee J, et al. (2007) Assessment of predictions submitted for the CASP7 domain prediction category. Proteins 69 Suppl 8: 137-151.

S3. Holbourn KP, Shone CC, Acharya KR. (2006) A family of killer toxins. exploring the mechanism of ADP-ribosylating toxins. FEBS J 273: 4579-4593.

S4. Cozzetto D, Giorgetti A, Raimondo D, Tramontano A. (2008) The evaluation of protein structure prediction results. Mol Biotechnol 39: 1-8.

S5. Giorgetti A, Raimondo D, Miele AE, Tramontano A. (2005) Evaluating the usefulness of protein structure models for molecular replacement. Bioinformatics 21 Suppl 2: ii72-6.

S6. Cheng J. (2007) DOMAC: An accurate, hybrid protein domain prediction server. Nucleic Acids Res 35: W354-6.

S7. Yeats C, Redfern OC, Orengo C. (2010) A fast and automated solution for accurately resolving protein domain architectures. Bioinformatics 26: 745-751.

S8. Tsuge H, Nagahama M, Oda M, Iwamoto S, Utsunomiya H, et al. (2008) Structural basis of actin recognition and arginine ADP-ribosylation by clostridium perfringens iota-toxin. Proc Natl Acad Sci U S A 105: 7399-7404.

S9. Sun J, Maresso AW, Kim JJ, Barbieri JT. (2004) How bacterial ADP-ribosylating toxins recognize substrates. Nat Struct Mol Biol 11: 868-876.

S10. Tian W, Skolnick J. (2003) How well is enzyme function conserved as a function of pairwise sequence identity? J Mol Biol 333: 863-882.
S11. Deng Q, Barbieri JT. (2008) Molecular mechanisms of the cytotoxicity of ADP-ribosylating toxins. Annu Rev Microbiol 62: 271-288.
S12. Finn RD, Tate J, Mistry J, Coggill PC, Sammut SJ, et al. (2008) The pfam protein families database. Nucleic Acids Res 36: D281-8.
S13. Barth H, Blocker D, Aktories K. (2002) The uptake machinery of clostridial actin ADP-ribosylating toxins--a cell delivery system for fusion proteins and polypeptide drugs. Naunyn Schmiedebergs Arch Pharmacol 366: 501-512.
S14. Fu ZQ, Guo M, Jeong BR, Tian F, Elthon TE, et al. (2007) A type III effector ADP-ribosylates RNA-binding proteins and quells plant immunity. Nature 447(7142): 284-288.
S15. Fieldhouse RJ, Merrill AR. (2008) Needle in the haystack: Structure-based toxin discovery. Trends Biochem Sci 33: 546-556.
S16. Armougom F, Moretti S, Poirot O, Audic S, Dumas P, et al. (2006) Expresso: Automatic incorporation of structural information in multiple sequence alignments using 3D-coffee. Nucleic Acids Res 34: W604-8.
S17. Gouet P, Robert X, Courcelle E. (2003) ESPript/ENDscript: Extracting and rendering sequence and 3D information from atomic structures of proteins. Nucleic Acids Res 31: 3320-3323.
S18. Shatsky M, Nussinov R, Wolfson HJ. (2004) A method for simultaneous alignment of multiple protein structures. Proteins 56: 143-156.

Supplementary Figures

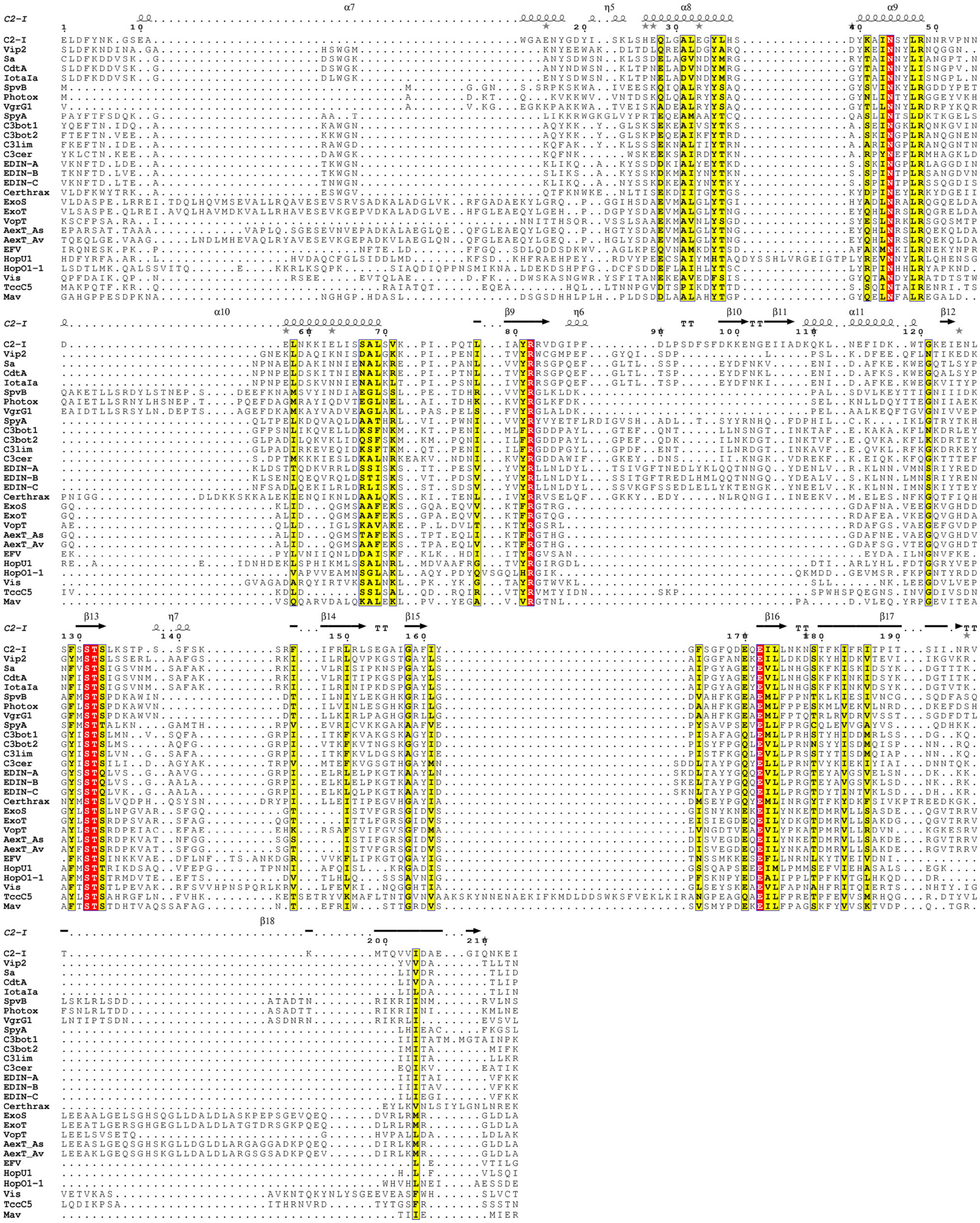

Figure S1. Multiple sequence alignment of ExoS-like, C2-like and C3-like toxin subgroups.
Multiple sequence alignments reveal conserved residues among the known and new ADPRT toxins within the ExoS-like, C2-like and C3-like toxin subgroups. In the alignment, red columns indicate identity and yellow columns indicate conserved positions. We built this alignment using 3D-Coffee [S16] and guided it with PDB files: SpvB (2GWMA), Iota (1GIQA), C3bot2 (1R45A), C3lim (3BW8A), C3bot1 (2BOVB), C2-I (2J3VA), C3stau2 (1OJQA), Vip2 (1QS1A) and SpvB (2GWLA). We visualized the alignment with ESPript [S17].

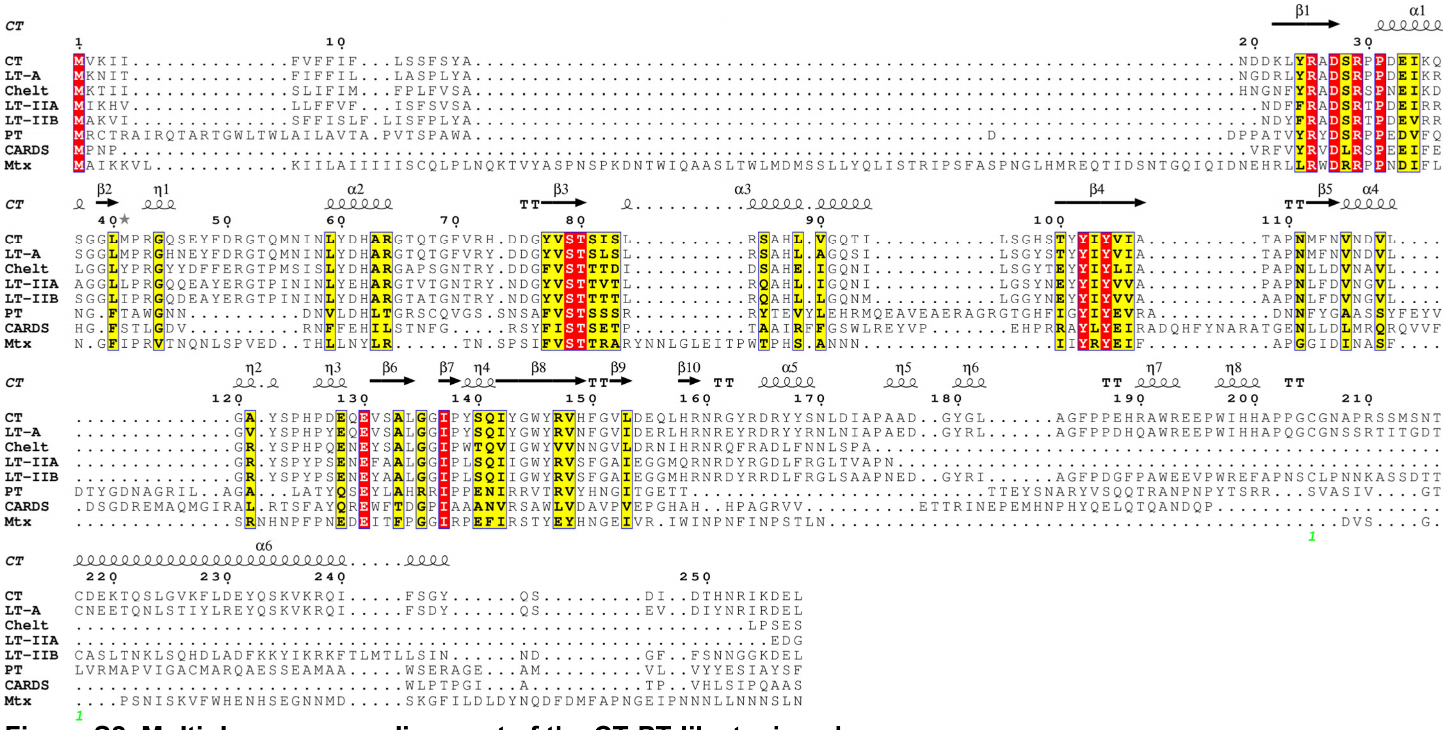

Figure S2. Multiple sequence alignment of the CT-PT-like toxin subgroup.

Multiple sequence alignments reveal conserved residues among the known and new ADPRT toxins within the CT-PT-like subgroup. In the alignment, red columns indicate identity and yellow columns indicate conserved positions. We built the alignment with 3D-Coffee [S16] and guided it with PDB files: CT (1S5EA), LT-IIB (1TIIA), Mtx (2VSAA), LT-A (1LT3A) and PT (1PTOA). The alignment was visualized by ESPript [S17].

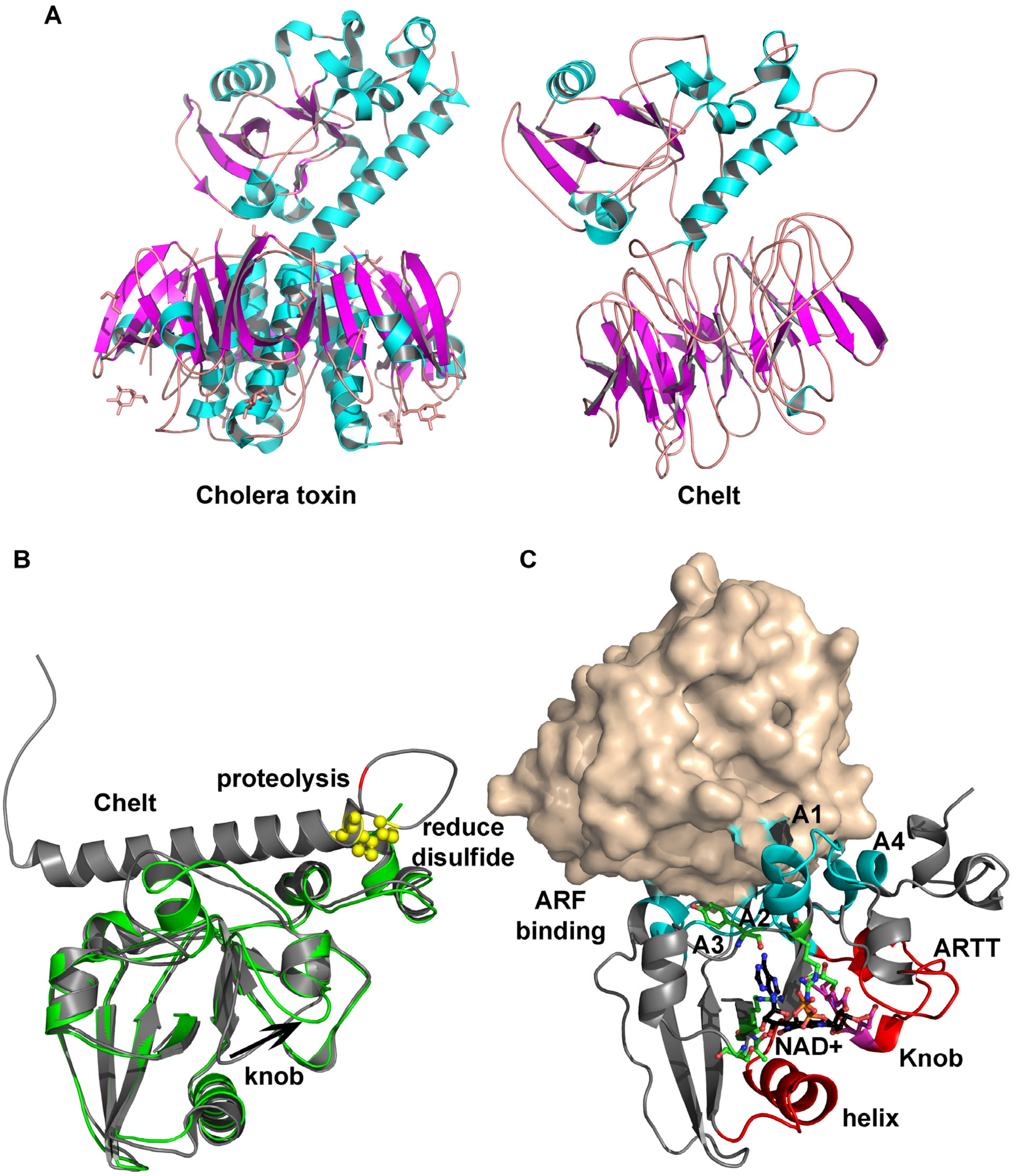

Figure S3. Chelt compared to cholera toxin and proposed Chelt-Arf complex.

(A) Cholera toxin (PDB 1S5D) (left) compared to Chelt (right). (B) Likeness to heat labile toxins suggest Chelt activation by reduction of the disulfide bond between C205 and C220, proteolysis at or near I215 (details unclear, due to a four amino acid deletion compared to LT-A between H214 and I215). (C) Comparison of the apo and NAD^+ bound models shows the knob region swinging on Arf binding, allowing NAD^+ to enter the active site. We show polar residues in green, positively charged residues in blue, negatively charged residues and target interaction loops in red, non-polar residues and the toxin scaffold in grey. Interaction with an ADP-ribosylating factor, perhaps ARF3, may be mediated by regions A1 (45-57), A2 (109-113), A3 (134-141) and A4 (167-182).

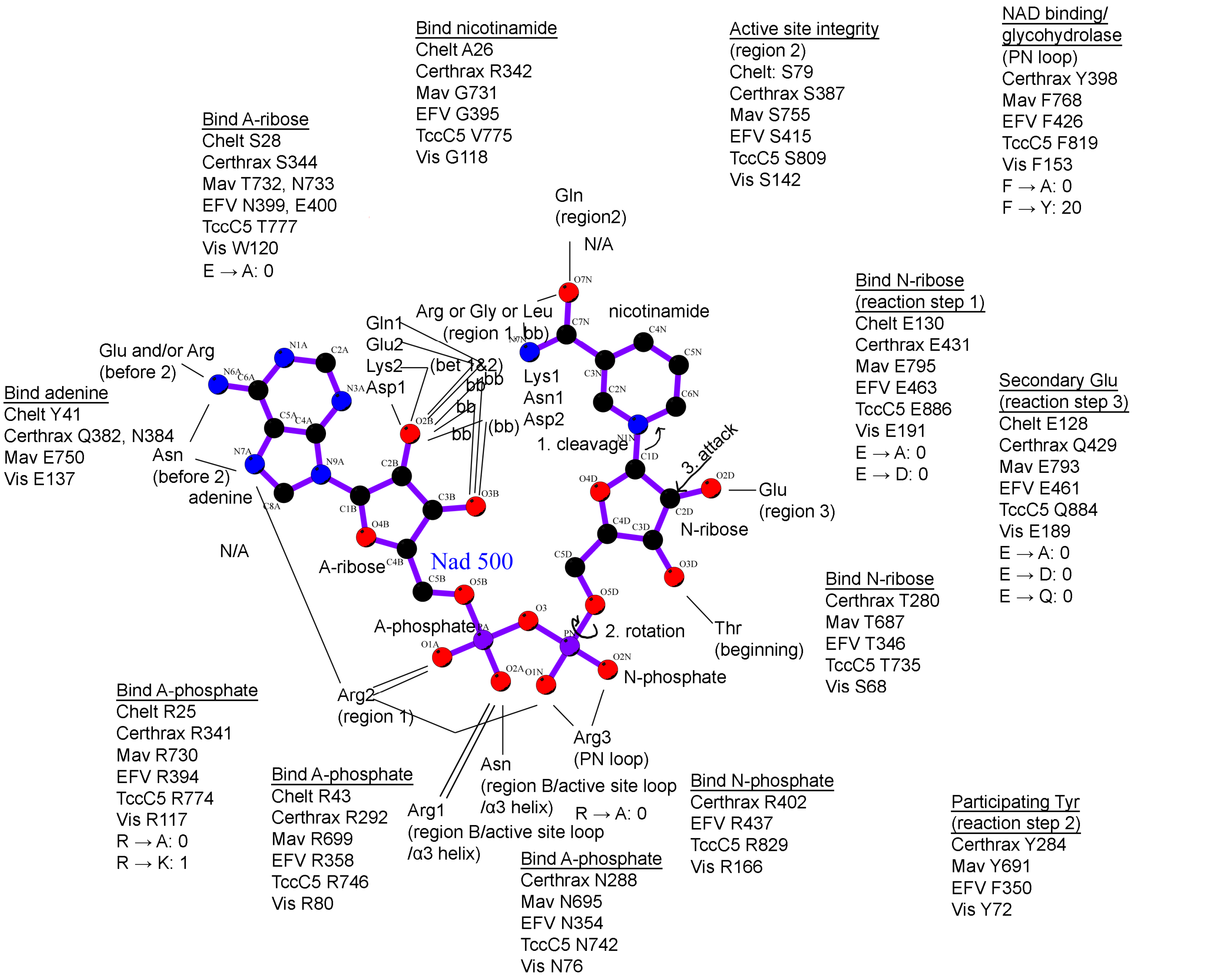

Figure S4. NAD$^+$ binding in the new toxins.

We substantiated toxin predictions using a digital NAD$^+$ binding test that reveals plausible NAD$^+$ binding mode for each new toxin. NAD$^+$ binding occurs through hydrogen bonds and hydrophobic interactions. H-bonds help position adenine (A), adenine-ribose (A-ribose), adenine- and nicotinamide-phosphate (A- and N-phosphate), nicotinamide-ribose (N-ribose) and nicotinamide. Most variation in NAD$^+$ binding occurs at the A-ribose, due to protein backbone H-bonding within a loop region instead of side chain H-bonding within a conserved secondary structure element. H-bonding is sparse at the nicotinamide ring. Interactions not involving NAD$^+$ are responsible for holding the catalytic Glu in place, orienting the secondary catalytic Glu or Gln and stiffening the active site. Hydrophobic interactions also help to hold the active site in place. Activity data from [S8] are shown.

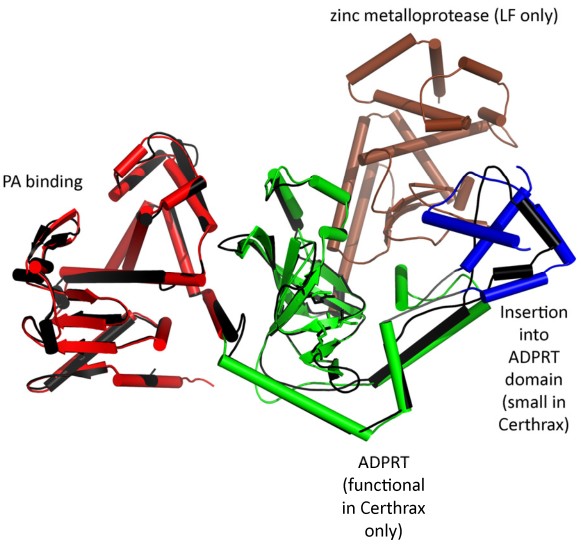

Figure S5. ***B. cereus*** **Certhrax compared to** ***B. anthracis*** **lethal factor.**

Certhrax model (black) compared to anthrax lethal factor (PDB 1J7N), superimposed by finding a common geometric core using MultiProt [S18]. Anthrax lethal factor PA binding domain (red), vestigial ADPRT domain (green), ADPRT domain insertion (blue) and catalytic metalloprotease domain (brown).

Supplementary Tables

Table S1. Percent Identity Matrix for the Catalytic Core of the Known and Putative Mono-ADP-Ribosylating Toxins

	AexT_A0FKE4	ExoS	AexT_Q93Q17	ExoT	VopT	Vip2Ac	Iotala	CdtA	Sa-	C2-I	SpvB	C3bot1	C3lim	C3bot2	EDIN-A	EDIN-B	EDIN-C	SpyA	HopU1	CT	LT-IIB	LT-IIA	LT-A	NarE	PT	CARDS	Mtx	VgrG1	EFV	Mav	Vis	TccC5	Chelt	Certhrax
AexT_A0FKE4	100	73	88	77	45	21	20	19	18	23	21	20	20	21	16	14	14	12	20	21	18	16	17	17	13	14	13	24	23	30	23	20	16	16
ExoS	73	100	71	76	40	25	22	19	18	23	21	22	26	24	18	16	18	12	20	22	21	21	21	20	11	13	17	24	26	30	26	23	17	16
AexT_Q93Q17	88	71	100	74	46	22	21	20	19	22	22	22	22	22	15	13	14	14	21	23	17	16	19	17	11	15	16	25	25	30	23	23	17	16
ExoT	77	76	74	100	43	24	21	21	20	23	22	25	25	24	16	19	19	14	19	23	18	18	19	21	10	17	14	23	21	33	20	20	17	16
VopT	45	40	46	43	100	22	22	24	23	16	23	16	18	18	15	16	13	14	22	18	15	13	17	22	13	14	16	27	22	27	26	22	17	18
Vip2Ac	21	25	22	24	22	100	42	42	41	30	28	35	30	31	33	29	29	25	21	19	14	13	19	19	21	13	19	29	25	24	18	19	13	29
Iotala	20	22	21	21	22	42	100	88	86	34	25	28	26	25	26	23	25	23	25	18	15	15	16	22	18	17	15	27	27	28	22	15	14	32
CdtA	19	19	20	21	24	42	88	100	91	33	25	27	24	25	26	23	23	25	25	16	14	14	14	20	18	17	17	26	27	27	25	16	13	34
Sa-	18	18	19	20	23	41	86	91	100	32	25	25	23	24	26	23	23	24	26	17	16	16	15	19	17	16	17	26	24	26	24	17	14	32
C2-I	23	23	22	23	16	30	34	33	32	100	24	21	21	22	19	18	20	19	24	15	13	12	15	17	16	12	14	21	24	24	28	16	10	26
SpvB	21	21	22	22	23	28	25	25	25	24	100	17	18	16	26	25	25	24	24	15	14	13	15	16	16	14	17	56	25	28	24	25	15	25
C3bot1	20	22	22	25	16	35	28	27	25	21	17	100	69	71	33	35	38	25	20	17	16	17	18	15	15	14	11	22	24	18	19	15	13	28
C3lim	20	26	22	25	18	30	26	24	23	21	18	69	100	73	31	35	36	21	18	18	15	16	19	18	16	13	13	21	29	22	19	16	15	30
C3bot2	21	24	22	24	18	31	25	25	24	22	16	71	73	100	30	33	34	22	18	16	14	15	17	16	14	14	14	19	28	25	22	15	12	28
EDIN-A	16	18	15	16	15	33	26	26	26	19	26	33	31	30	100	83	69	29	16	15	12	12	16	9	15	17	11	31	21	21	16	18	12	28
EDIN-B	14	16	13	19	16	29	23	23	23	18	25	35	35	33	83	100	76	29	18	13	11	10	13	12	15	16	13	29	24	21	17	18	12	28
EDIN-C	14	18	14	19	13	29	25	23	23	20	25	38	36	34	69	76	100	29	21	13	12	13	13	12	15	14	14	29	24	20	16	16	10	29
SpyA	12	12	14	14	14	25	23	25	24	19	24	25	21	22	29	29	29	100	24	15	19	19	15	13	15	13	19	23	23	29	18	20	21	23
HopU1	20	20	21	19	22	21	25	25	26	24	24	20	18	18	16	18	21	24	100	16	17	18	16	21	14	13	23	25	24	30	28	11	19	20
CT	21	22	23	23	18	19	18	16	17	15	15	17	18	16	15	13	13	15	16	100	64	64	89	24	28	20	26	12	19	17	15	15	59	14
LT-IIB	18	21	17	18	15	14	15	14	16	13	14	16	15	14	12	11	12	19	17	64	100	88	65	25	28	19	25	12	14	18	16	10	63	14
LT-IIA	16	21	16	18	13	13	15	14	16	12	13	17	16	15	12	10	13	19	18	64	88	100	64	24	25	20	26	12	14	17	15	10	63	15
LT-A	17	21	19	19	17	19	16	14	15	15	15	18	19	17	16	13	13	15	16	89	65	64	100	27	27	21	27	12	15	16	15	13	61	15
NarE	17	20	17	21	22	19	22	20	19	17	16	15	18	16	9	12	12	13	21	24	25	24	27	100	18	18	19	14	16	21	26	12	23	14
PT	13	11	11	10	13	21	18	18	17	16	16	15	16	14	15	15	15	15	14	28	28	25	27	18	100	30	28	18	19	16	17	24	22	17
CARDS	14	13	15	17	14	13	17	17	16	12	14	14	13	14	17	16	14	13	13	20	19	20	21	18	30	100	21	17	12	16	15	15	21	17
Mtx	13	17	16	14	16	19	15	17	17	14	17	11	13	14	11	13	14	19	23	26	25	26	27	19	28	21	100	17	13	21	18	16	29	13
VgrG1	24	24	25	23	27	29	27	26	26	21	56	22	21	19	31	29	29	23	25	12	12	12	12	14	18	17	17	100	25	24	26	22	16	27
EFV	23	26	25	21	22	25	27	27	24	24	25	24	29	28	21	24	24	23	24	19	14	14	15	16	19	12	13	25	100	27	29	16	19	27
Mav	30	30	30	33	27	24	28	27	26	24	28	18	22	25	21	21	20	29	30	17	18	17	16	21	16	16	21	24	27	100	33	20	22	22
Vis	23	26	23	20	26	18	22	25	24	28	24	19	19	22	16	17	16	18	28	15	16	15	15	26	17	15	18	26	29	33	100	17	15	22
TccC5	20	23	23	20	22	19	15	16	17	16	25	15	16	15	18	18	16	20	11	15	10	10	13	12	24	15	16	22	16	20	17	100	12	16
Chelt	16	17	17	17	17	13	14	13	14	10	15	13	15	12	12	12	10	21	19	59	63	63	61	23	22	21	29	16	19	22	15	12	100	15
Certhrax	16	16	16	16	18	29	32	34	32	26	25	28	30	28	28	28	29	23	20	14	14	15	15	14	17	17	13	27	27	22	22	16	15	100

Legend

Midnight zone	0	to	24
Twilight zone	25	to	34
Safe zone	35	to	100

Notes

mART core is approximately 100 residues long

Alignment constructed with SPEM using sequence profiles and predicted secondary structure

Matrix constructed with ClustalX 1.83

Table S2. New ADPRT toxin model quality

Name	Template	Predicted GDT-TS[a]	Estimated RMSD[b]	TM-score[c]	Model quality[d]
**Full-length**					
Chelt	1lt4, 2vdr[e]	45.67	-	-	High
Certhrax	1j7n, 1xfu, 1qs1	36.40	6.8	0.74	High
Mav toxin	1wa8, 1ciy, ADPRT	28.27	-	-	Fair
EFV toxin	1u4q, ADPRT	4.52	-	-	Poor
TccC5	1k32, 2fkj, 2ecf, 2pn5, 2fgz, 1n1m	29.18	12.1	0.55	Fair
Vis toxin	single domain – see catalytic domain below				
**Catalytic domain (NAD$^+$-bound)**					
Chelt	2a5f	45.14	4.83	0.29	Excellent
Certhrax	1ojz	50.85	1.07	0.92	Good
	2a9k	49.36	6.38	0.26	
	1qs2	48.53	0.57	0.48	
	1giq	48.18	0.43	0.49	
	2wn7	48.18	0.54	0.52	
	1og3	45.24	1.81	0.75	
	2gwl	44.93	2.15	0.83	
Mav toxin	1ojz	44.46	1.22	0.74	Good
	2a9k	44.39	5.75	0.26	
	1qs2	42.88	1.56	0.42	
	1giq	44.7	1.51	0.35	
	2wn7	46.51	1.43	0.43	
	1og3	43.99	1.97	0.73	
	2gwl	46.14	1.32	0.69	
EFV toxin	1ojz	49.3	0.93	0.82	Good
	2a9k	48.22	5.13	0.28	
	1qs2	46.66	2.07	0.41	
	1giq	45.26	0.93	0.40	
	2wn7	45.59	1.52	0.42	
	1og3	46.47	1.89	0.73	
	2gwl	46.38	0.98	0.68	
TccC5	1ojz	45.48	1.92	0.82	Fair
	2a9k	39.91	5.38	0.31	
	1qs2	46.25	1.25	0.40	
	1giq	47.17	1.24	0.42	
	2wn7	35.07	3.58	0.36	
	1og3	32.32	3.69	0.51	
	2gwl	47.69	1.67	0.70	
Vis toxin	1ojz	44.89	0.83	0.80	Good
	2a9k	43.94	5.76	0.29	
	1qs2	46.77	1.29	0.43	
	1giq	47.54	1.25	0.48	
	2wn7	42.42	2.29	0.46	
	1og3	43.81	0.83	0.88	
	2gwl	41.02	0.93	0.84	

[a] Global distance test total score (GDT-TS) counts Cα pairs that have a distance <1, 2, 4, 8 Å after overlap. Larger values are better. Values >40 imply correct fold and values >84 imply reliable use in molecular replacement. The low GDT-TS for EFV toxin full-length may reflect our inability to predict orientation of the component domains.
[b] Compared to the native structure; smaller is better.
[c] Template modeling score (TM-score) values greater than 0.5 suggests correct topology.
[d] For catalytic domains, based on sequence identity (<=20% “poor”, 21-25% “fair”, 26-30% “good”, 31-35% “high”, >=36% “excellent”)
[e] Better coverage than 2bwr

Table S3. Summary statistics for model geometry

Full-length							
	Ideal	Chelt	Certhrax	Mav	EFV	TccC5	Vis
Rotamer outliers (%)	<1	5.28	6.51	2.67	4.40	4.91	see below
Ramachandran outliers	<0.2	2.41	5.70	2.67	1.65	7.48	
Ramachandran favored	>98	88.81	87.55	87.97	90.72	84.38	
Cβ deviations >0.25Å	0	7	35	4	6	7	
Residues with bad bonds	<1	0.00	0.00	0.00	0.00	34.65	
Residues with bad angles	<0.5	1.54	5.88	0.85	2.26	34.43	
**Catalytic domain (NAD^+-bound)**							
	Ideal	Chelt	Certhrax	Mav	EFV	TccC5	Vis
Rotamer outliers (%)	<1	4.52	13.15	2.88	9.09	7.14	11.62
Ramachandran outliers	<0.2	0.55	4.74	4.85	4.40	5.92	4.80
Ramachandran favored	>98	98.36	87.07	88.48	85.71	85.21	89.52
Cβ deviations >0.25Å	0	1	10	3	4	7	5
Residues with bad bonds	<1	0.00	0.00	0.00	0.00	3.51	0.87
Residues with bad angles	<0.5	0.54	2.56	2.40	4.89	9.36	3.90

Dataset S1

Chelt

target name	len	blast	pdb-psi	pdb	scop	cath	interpret	rank	3djury
A2PU44	601	1E-66	2E-80	1lt4A	d.166.1.1	3.90.210.10	ADPRT toxin	1	178.12
A2PU44_1-300	300	2E-65	3E-78	1lt4A	d.166.1.1	3.90.210.10	ADPRT toxin	1	158
A2PU44_1-350	350	3E-65	5E-76	1lt4A	d.166.1.1	3.90.210.10	ADPRT toxin	1	157.75
A2PU44_1-600	600	4E-65	5E-84	1lt4A	d.166.1.1	3.90.210.10	ADPRT toxin	1	156.5
A2PU44_1-250	250	8E-66	4E-78	1lt4A	d.166.1.1	3.90.210.10	ADPRT toxin	1	152.75
A2PU44_1-400	400	3E-65	4E-73	1lt4A	d.166.1.1	3.90.210.10	ADPRT toxin	1	152.75
A2PU44_1-550	550	4E-65	4E-53	1lt4A	d.166.1.1	3.90.210.10	ADPRT toxin	1	150.25
A2PU44_1-450	450	5E-65	1E-57	1lt4A	d.166.1.1	3.90.210.10	ADPRT toxin	1	147.75
A2PU44_1-500	500	5E-65	6E-53	1s5eA	d.166.1.1	3.90.210.10	ADPRT toxin	1	143.25
A2PU44_1-216	216	4E-64	1E-69	1lt4A	d.166.1.1	3.90.210.10	ADPRT toxin	1	141.25
A2PU44_1-200	200	2E-60	3E-64	1xtcA	d.166.1.1	3.90.210.10	ADPRT toxin	1	134
A2PU44_1-179	179	2E-54	3E-52	1xtcA	d.166.1.1	3.90.210.10	ADPRT toxin	1	131
A2PU44_101-250	150	2E-37	1E-52	1lt3A	d.166.1.1	3.90.210.10	ADPRT toxin	1	98.75
A2PU44_1-150	150	3E-47	4E-43	1xtcA	d.166.1.1	3.90.210.10	ADPRT toxin	1	96.5
A2PU44_101-200	100	3E-31	3E-39	1ltsA	d.166.1.1	3.90.210.10	ADPRT toxin	1	74.75
A2PU44_51-150	100	5E-38	7E-39	1tiiA	d.166.1.1	3.90.210.10	ADPRT toxin	1	74.25
A2PU44_151-250	100	1E-16	2E-45	1lt3A	d.166.1.1	3.90.210.10	ADPRT toxin	1	62.5
A2PU44_1-100	100	2E-24	5E-38	1xtcA	d.166.1.1	3.90.210.10	ADPRT toxin	1	59
A2PU44_101-175	75	2E-24	7E-27	1tiiA	d.166.1.1	3.90.210.10	ADPRT toxin	1	55.5
A2PU44_51-125	75	5E-27	2E-33	1tiiA	d.166.1.1	3.90.210.10	ADPRT toxin	1	55.5
A2PU44_151-225	75	2E-15	3E-32	1tiiA	d.166.1.1	3.90.210.10	ADPRT toxin	1	46
A2PU44_180-240	61	3E-09	7E-29	1s5eA	d.166.1.1	3.90.210.10	ADPRT toxin	1	42.33
A2PU44_1-75	75	5E-15	1E-25	1ltiA	d.166.1.1	3.90.210.10	ADPRT toxin	1	40.5
A2PU44_101-150	50	9E-17	1E-18	1tiiA	d.166.1.1	3.90.210.10	ADPRT toxin	1	37.5
A2PU44_76-125	50	3E-15	6E-17	1tiiA	d.166.1.1	3.90.210.10	ADPRT toxin	1	37.5
A2PU44_151-200	50	8E-11	5E-25	1xtcA	d.166.1.1	3.90.210.10	ADPRT toxin	1	37.5
A2PU44_126-175	50	1E-12	4E-22	1tiiA	d.166.1.1	3.90.210.10	ADPRT toxin	1	37
A2PU44_51-100	50	5E-15	9E-20	1tiiA	d.166.1.1	3.90.210.10	ADPRT toxin	1	36.75
A2PU44_251-550	300	0.45	4E-15	2bwmA	b.69.8	2.130.10	lectin	2	152
A2PU44_301-601	301	0.27	5E-14	2bwmA	b.69.8	2.130.10	lectin	2	142
A2PU44_241-601	361	0.67	7E-17	2bwrA	b.69.8	2.130.10	lectin	2	141.25
A2PU44_290-601	312	0.26	2E-14	2bwmA	b.69.8	2.130.10	lectin	2	138.33
A2PU44_201-450	250	0.12	3E-18	3ijeA	b.69.8, b.1.15	2.130.10, 2.60.40	protein binding	2	114
A2PU44_351-601	251	0.21	4E-31	2bwrA	b.69.8	2.130.10	lectin	2	99.75
A2PU44_301-500	200	0.21	3E-14	2bwmA	b.69.8	2.130.10	lectin	2	93.75
A2PU44_151-350	200	4E-16	5E-35	2bwmA	b.69.8	2.130.10	lectin	2	75.5
A2PU44_401-601	201	0.16	1.9	3ijeA	b.69.8, b.1.15	2.130.10, 2.60.40	protein binding	2	73.5
A2PU44_218-376	159	0.24	8E-08	2bwrA	b.69.8	2.130.10	lectin	2	56.25
A2PU44_201-350	150	0.032	1.5	2bwmA	b.69.8	2.130.10	lectin	2	53.5
A2PU44_401-550	150	0.12	0.84	2bwrA	b.69.8	2.130.10	lectin	2	51.25
A2PU44_301-450	150	0.42	2E-06	3ijeA	b.69.8, b.1.15	2.130.10, 2.60.40	protein binding	2	51
A2PU44_251-350	100	0.081	0.77	3ijeA	b.69.8, b.1.15	2.130.10, 2.60.40	protein binding	2	30
A2PU44_451-601	151	1.7	0.027	1svpA	b.47.1.3	2.40.10.10, 2.40.10.10	viral protein		48.25
A2PU44_501-600	100	0.93	0.15	1c76A	d.15.5.1	3.10.20.130	hydrolase		29.75
A2PU44_301-400	100	0.28	0.021	1jv2A	b.1.15.1, b.1.15.1, b.1.15.1	2.130.10.130, 2.60.40.1460, 2.60.40.1510	cell adhesion		24
A2PU44_502-601	100	1	0.2	1nezG	b.1.1.1	2.60.40.10	immune system		18.25
A2PU44_501-601	101	0.98	0.17	3b9kA	b.1.1	2.60.40.10	immune system		18.25
A2PU44_451-550	100	1	0.35	2erjD	a.26.1.2	1.20.1250.10	immune system / cytokine		17.75
A2PU44_401-500	100	0.073	0.31	3kivA	g.14.1.1	2.40.20.10	kringle		17.75
A2PU44_377-501	125	0.086	1.7	1n02A	b.89.1.1	2.30.60.10	virus/viral protein		16
A2PU44_501-575	75	0.67	0.32	1nyqA	c.51.1.1, d.15.10.1, d.67.1.1	3.40.50, 3.10.20, ?	ligase		16
A2PU44_351-425	75	0.89	1.5	1c8dA	b.121.5.2	2.170.30.10	viral protein		15.5
A2PU44_201-275	75	2.3	1.7	3lkeA	c.14.1	3.90.226	lyase		13.25
A2PU44_251-325	75	0.43	0.19	1xq4A	b.1.23.1	2.60.40.1470	structural genomics		12.5
A2PU44_201-300	100	1.8	1.7	1qpxA	b.1.11.1, b.7.2.1,	2.60.40.360, 2.60.40.1070	chaperone		12.25

A2PU44_201-250	50	3	2	2qadA	d.172.1	2.170.40	viral protein/immune		12.25
A2PU44_351-450	100	1.4	0.87	1c8dA	b.121.5.2	2.170.30.10	viral protein		12
A2PU44_351-400	50	0.79	2	2htyA	b.68.1	2.120.10.10	hydrolase		12
A2PU44_451-525	75	1.1	0.21	3ed8A	d.22.1	2.40.155.10	luminescent protein		12
A2PU44_251-300	50	2.4	2.7	1j7mA	g.14.1.2	2.10.10.10	hydrolase		11.5
A2PU44_226-275	50	4	1.8	2jx5A	d.15.1	3.10.20	ribosomal protein		11.5
A2PU44_526-575	50	10	0.75	3gwjA	b.1.18, a.86.1, a.85.1	2.70, 2.60.40; 1.10.1280; 1.20.1370	oxygen transport		11.25
A2PU44_551-600	50	6.5	2	3js9A	d.58.6	3.30.70	transferase		10.5
A2PU44_551-601	51	6.7	2	3js9A	d.58.6	3.30.70	transferase		10.5
A2PU44_501-550	50	1.2	0.32	1qf6A	c.51.1.1, d.15.10.1, d.67.1.1	3.10.20.30, 3.30.980.10, 3.30.54.20	ligase/rna		10.25
A2PU44_301-375	75	0.87	0.01	1v9hA	d.124.1.1	3.90.730.10	hydrolase		10.25
A2PU44_326-375	50	0.94	0.011	3eswA	d.3.1	3.90.70, 3.90.260, 3.40.532, 3.90.1360, 3.40.395	hydrolase		10.25
A2PU44_376-425	50	5.8	6	1e8pA	g.55.1.1	3.90.1220.10	cellulose docking domain		10
A2PU44_276-325	50	1.5	0.35	2e56A	b.1.18	2.70, 2.60.40	lipid binding		10
A2PU44_241-289	49	2.4	1.5	1mr8A	a.39.1.2	1.10.238.10	metal transport		7

Certhrax

target name	len	blast	pdb-psi	pdb	scop	cath	interpret	rank	3djury
Q4MV79_1-450	450	7E-35	1E-92	1j7nA	d.92.1.14, d.92.1.14, d.166.1.1	3.40.390.10, 3.90.176.10, 1.10.2030.10	anthrax lethal factor	1	239
Q4MV79_1-400	400	4E-35	1E-92	1j7nA	d.92.1.14, d.92.1.14, d.166.1.1	3.40.390.10, 3.90.176.10, 1.10.2030.10	anthrax lethal factor	1	222.75
Q4MV79_1-450	420	6E-35	2E-95	1j7nA	d.92.1.14, d.92.1.14, d.166.1.1	3.40.390.10, 3.90.176.10, 1.10.2030.10	anthrax lethal factor	1	222
Q4MV79	476	1E-39	6E-91	1j7nA	d.92.1.14, d.92.1.14, d.166.1.1	3.40.390.10, 3.90.176.10, 1.10.2030.10	anthrax lethal factor	1	217.25
Q4MV79_1-350	350	4E-35	1E-100	1j7nA	d.92.1.14, d.92.1.14, d.166.1.1	3.40.390.10, 3.90.176.10, 1.10.2030.10	anthrax lethal factor	1	200.75
Q4MV79_1-300	300	5E-35	3E-93	1j7nA	d.92.1.14, d.92.1.14, d.166.1.1	3.40.390.10, 3.90.176.10, 1.10.2030.10	anthrax lethal factor	1	189.25
Q4MV79_1-250	250	1E-34	4E-83	1j7nA	d.92.1.14, d.92.1.14, d.166.1.1	3.40.390.10, 3.90.176.10, 1.10.2030.10	anthrax lethal factor	1	170.75
Q4MV79_1-254	254	1E-34	3E-83	1PWUA	d.92.1.14, d.92.1.14, d.166.1.1	3.40.390.10, 3.90.176.10, 1.10.2030.10	anthrax lethal factor	1	136
Q4MV79_1-200	200	6E-27	2E-64	1j7nA	d.92.1.14, d.92.1.14, d.166.1.1	3.40.390.10, 3.90.176.10, 1.10.2030.10	anthrax lethal factor	1	135
Q4MV79_1-150	150	1E-21	2E-53	1j7nA	d.92.1.14, d.92.1.14, d.166.1.1	3.40.390.10, 3.90.176.10, 1.10.2030.10	anthrax lethal factor	1	109.25
Q4MV79_101-250	150	9E-15	3E-41	1j7nA	d.92.1.14, d.92.1.14, d.166.1.1	3.40.390.10, 3.90.176.10, 1.10.2030.10	anthrax lethal factor	1	95.25
Q4MV79_151-350	200	5E-12	9E-46	1j7nA	d.92.1.14, d.92.1.14, d.166.1.1	3.40.390.10, 3.90.176.10, 1.10.2030.10	anthrax lethal factor	1	79.25
Q4MV79_1-100	100	1E-15	4E-36	1j7nA	d.92.1.14, d.92.1.14, d.166.1.1	3.40.390.10, 3.90.176.10, 1.10.2030.10	anthrax lethal factor	1	74.5
Q4MV79_51-150	100	1E-09	1E-31	1j7nA	d.92.1.14, d.92.1.14, d.166.1.1	3.40.390.10, 3.90.176.10, 1.10.2030.10	anthrax lethal factor	1	71.5
Q4MV79_151-250	100	1E-11	6E-33	1j7nA	d.92.1.14, d.92.1.14, d.166.1.1	3.40.390.10, 3.90.176.10, 1.10.2030.10	anthrax lethal factor	1	70.75
Q4MV79_1-75	75	5E-10	1E-24	1pwuA	d.92.1.14, d.92.1.14, d.166.1.1	3.40.390.10, 3.90.176.10, 1.10.2030.10	anthrax lethal factor	1	56.25
Q4MV79_101-200	100	2E-07	6E-27	1pwuA	d.92.1.14, d.92.1.14, d.166.1.1	3.40.390.10, 3.90.176.10, 1.10.2030.10	anthrax lethal factor	1	55.25
Q4MV79_151-225	75	2E-11	9E-28	1j7nA	d.92.1.14, d.92.1.14, d.166.1.1	3.40.390.10, 3.90.176.10, 1.10.2030.10	anthrax lethal factor	1	53.5
Q4MV79_51-125	75	6E-09	6E-24	1j7nA	d.92.1.14, d.92.1.14, d.166.1.1	3.40.390.10, 3.90.176.10, 1.10.2030.10	anthrax lethal factor	1	52.25
Q4MV79_201-275	75	0.005	0.004	1j7nA	d.92.1.14, d.92.1.14, d.166.1.1	3.40.390.10, 3.90.176.10, 1.10.2030.10	anthrax lethal factor	1	38.5
Q4MV79_176-225	50	2E-07	1E-19	1j7nA	d.92.1.14, d.92.1.14, d.166.1.1	3.40.390.10, 3.90.176.10, 1.10.2030.10	anthrax lethal factor	1	37.5
Q4MV79_26-75	50	0.000001	1E-16	1j7nA	d.92.1.14, d.92.1.14, d.166.1.1	3.40.390.10, 3.90.176.10, 1.10.2030.10	anthrax lethal factor	1	37.5
Q4MV79_1-50	50	7E-07	3E-15	1j7nA	d.92.1.14, d.92.1.14, d.166.1.1	3.40.390.10, 3.90.176.10, 1.10.2030.10	anthrax lethal factor	1	37.5
Q4MV79_51-100	50	0.001	0.0002	1j7nA	d.92.1.14, d.92.1.14, d.166.1.1	3.40.390.10, 3.90.176.10, 1.10.2030.10	anthrax lethal factor	1	36.25
Q4MV79_201-250	50	0.008	0.005	1j7nA	d.92.1.14, d.92.1.14, d.166.1.1	3.40.390.10, 3.90.176.10, 1.10.2030.10	anthrax lethal factor	1	24.5
Q4MV79_151-200	50	0.0007	0.0003	1j7nA	d.92.1.14, d.92.1.14, d.166.1.1	3.40.390.10, 3.90.176.10, 1.10.2030.10	anthrax lethal factor	1	23.5
Q4MV79_76-125	50	0.00001	0.000005	1j7nA	d.92.1.14, d.92.1.14, d.166.1.1	3.40.390.10, 3.90.176.10, 1.10.2030.10	anthrax lethal factor	1	23
Q4MV79_251-476	226	4E-18	3E-36	1giqA	d.166.1.1, d.166.1.1	3.90.176.10, 3.90.176.10,	ADPRT toxin	2	143
Q4MV79_201-450	250	9E-20	3E-35	1giqA	d.166.1.1, d.166.1.1	3.90.176.10, 3.90.176.10,	ADPRT toxin	2	140.25
Q4MV79_301-476	176	5E-14	1E-33	2wn4A	d.166.1.1	3.90.176, 3.90.175, 3.90.210	ADPRT toxin	2	112
Q4MV79_301-450	150	4E-14	7E-32	1giqA	d.166.1.1, d.166.1.1	3.90.176.10, 3.90.176.10,	ADPRT toxin	2	104.5
Q4MV79_351-476	126	3E-09	2E-22	2wn4A	d.166.1.1	3.90.176, 3.90.175, 3.90.210	ADPRT toxin	2	76.5
Q4MV79_201-350	150	6E-11	4E-23	1qs1A	d.166.1.1, d.166.1.1	3.90.176.10, 3.90.176.10,	ADPRT toxin	2	70.5
Q4MV79_301-400	100	0.00001	2E-26	3bw8A	d.166.1.1	3.90.176.10	ADPRT toxin	2	69
Q4MV79_351-450	100	3E-09	8E-22	1qs1A	d.166.1.1, d.166.1.1	3.90.176.10, 3.90.176.10,	ADPRT toxin	2	66
Q4MV79_251-350	100	1E-10	1E-22	1giqA	d.166.1.1, d.166.1.1	3.90.176.10, 3.90.176.10,	ADPRT toxin	2	64
Q4MV79_390-476	87	0.00006	1E-18	3buzA	d.166.1.1, d.166.1.1	3.90.176.10, 3.90.176.10,	ADPRT toxin	2	55.5
Q4MV79_301-375	75	0.0008	0.003	2wn4A	d.166.1.1	3.90.176, 3.90.175, 3.90.210	ADPRT toxin	2	48.25
Q4MV79_351-425	75	0.002	4E-19	2a78B	d.166.1.1	3.90.176.10	ADPRT toxin	2	47.75
Q4MV79_401-475	75	0.000003	2E-15	1qs1A	d.166.1.1, d.166.1.1	3.90.176.10, 3.90.176.10,	ADPRT toxin	2	46.5
Q4MV79_401-476	76	0.000003	2E-15	1qs1A	d.166.1.1, d.166.1.1	3.90.176.10, 3.90.176.10,	ADPRT toxin	2	46
Q4MV79_201-300	100	0.00002	2E-16	1giqA	d.166.1.1, d.166.1.1	3.90.176.10, 3.90.176.10,	ADPRT toxin	2	43.5
Q4MV79_251-325	75	0.00003	2E-13	1giqA	d.166.1.1, d.166.1.1	3.90.176.10, 3.90.176.10,	ADPRT toxin	2	42.5
Q4MV79_340-389	50	0.8	0.16	2bovB	d.166.1.1	3.90.176.10	ADPRT toxin	2	40.33
Q4MV79_251-300	50	0.00006	1E-13	1qs1A	d.166.1.1, d.166.1.1	3.90.176.10, 3.90.176.10,	ADPRT toxin	2	36.75
Q4MV79_401-450	50	0.000005	8E-15	1qs1A	d.166.1.1, d.166.1.1	3.90.176.10, 3.90.176.10,	ADPRT toxin	2	36.5
Q4MV79_326-375	50	0.39	0.1	1giqA	d.166.1.1, d.166.1.1	3.90.176.10, 3.90.176.10,	ADPRT toxin	2	36.25
Q4MV79_376-425	50	0.089	0.05	2wn4A	d.166.1.1	3.90.176, 3.90.175, 3.90.210	ADPRT toxin	2	36
Q4MV79_351-400	50	0.4	0.055	1giqA	d.166.1.1, d.166.1.1	3.90.176.10, 3.90.176.10,	ADPRT toxin	2	34.5
Q4MV79_301-350	50	0.002	0.007	2j3vA	d.166.1.1	3.90.176, 3.90.175, 3.90.210	ADPRT toxin	2	24

Q4MV79_276-325	50	4.9	1.6	3bw8A	d.166.1.1	3.90.176.10	ADPRT toxin	2	21.25
Q4MV79_426-475	50	1.5	1.4	2a78B	d.166.1.1	3.90.176.10	ADPRT toxin	2	10.5
Q4MV79_101-175	75	0.003	0.0008	2dhrA	c.37.1, a.269.1	3.40.50, 3.40.850, 3.40.1140, 1.10.8; ?	hydrolase		25.25
Q4MV79_101-150	50	1.3	0.64	1r7rA	b.52.2.3, c.37.1.20, c.37.1.20	2.40.40.20, 3.10.330.10, 3.40.50.300	transport protein		21.75

Mav toxin

target name	len	blast	pdb-psi	pdb	scop	cath	interpret	Rank	3djury
A0QLI5_612-825	214	0.002	8E-43	1gxyA	d.166.1.3	3.90.176.10	ADPRT toxin	1	125.75
A0QLI5_640-825	186	0.0006	8E-31	1giqA	d.166.1.1, d.166.1.1	3.90.176.10, 3.90.176.10	ADPRT toxin	1	124.83
A0QLI5_601-825	225	0.003	9E-43	2j3vA	d.166.1.1	3.90.176, 3.90.175, 3.90.210	ADPRT toxin	1	120.5
A0QLI5_675-825	151	0.0002	2E-28	2wn4A	d.166.1.1	3.90.176, 3.90.175, 3.90.210	ADPRT toxin	1	107.5
A0QLI5_676-825	150	0.0002	7E-28	2wn4A	d.166.1.1	3.90.176, 3.90.175, 3.90.210	ADPRT toxin	1	107.5
A0QLI5_676-825	150	0.0002	3E-25	2wn4A	d.166.1.1	3.90.176, 3.90.175, 3.90.210	ADPRT toxin	1	106
A0QLI5_515-825	311	0.008	7E-21	3buzA	d.166.1.1, d.166.1.1	3.90.176.10, 3.90.176.10	ADPRT toxin	1	103.25
A0QLI5_531-825	295	0.005	5E-34	2bovB	d.166.1.1	3.90.176.10	ADPRT toxin	1	101
A0QLI5_451-825	375	0.017	9E-23	3bw8A	d.166.1.1	3.90.176.10	ADPRT toxin	1	99
A0QLI5_601-800	200	0.014	2E-38	1gxyA	d.166.1.3	3.90.176.10	ADPRT toxin	1	97.5
A0QLI5_501-825	325	0.009	6E-19	2wn4A	d.166.1.1	3.90.176, 3.90.175, 3.90.210	ADPRT toxin	1	97.25
A0QLI5_696-825	130	0.0001	7E-25	2wn4A	d.166.1.1	3.90.176, 3.90.175, 3.90.210	ADPRT toxin	1	91
A0QLI5_501-800	300	0.03	3E-21	2wn4A	d.166.1.1	3.90.176, 3.90.175, 3.90.210	ADPRT toxin	1	90.75
A0QLI5_383-825	443	0.025	8E-20	2a78B	d.166.1.1	3.90.176.10	ADPRT toxin	1	81
A0QLI5_701-825	125	0.0001	5E-23	2wn4A	d.166.1.1	3.90.176, 3.90.175, 3.90.210	ADPRT toxin	1	79.33
A0QLI5_401-825	425	0.02	4E-19	2bovB	d.166.1.1	3.90.176.10	ADPRT toxin	1	79
A0QLI5_701-800	100	0.0007	3E-25	1qs1A	d.166.1.1, d.166.1.1	3.90.176.10, 3.90.176.10	ADPRT toxin	1	70.75
A0QLI5_292-825	534	0.033	2E-14	2wn4A	d.166.1.1	3.90.176, 3.90.175, 3.90.210	ADPRT toxin	1	69
A0QLI5_728-825	98	0.04	8E-19	1qs1A	d.166.1.1, d.166.1.1	3.90.176.10, 3.90.176.10	ADPRT toxin	1	66.75
A0QLI5_736-825	90	0.042	5E-17	3buzA	d.166.1.1, d.166.1.1	3.90.176.10, 3.90.176.10	ADPRT toxin	1	63.75
A0QLI5	825	0.008	2E-20	2bovB	d.166.1.1	3.90.176.10	ADPRT toxin	1	59.8
A0QLI5_651-750	100	0.43	0.000004	2a78B	d.166.1.1	3.90.176.10	ADPRT toxin	1	59.75
A0QLI5_601-750	150	0.96	1E-07	2wn4A	d.166.1.1	3.90.176, 3.90.175, 3.90.210	ADPRT toxin	1	56.75
A0QLI5_751-825	75	0.023	2E-16	2wn4A	d.166.1.1	3.90.176, 3.90.175, 3.90.210	ADPRT toxin	1	52.5
A0QLI5_701-775	75	0.37	0.00007	2wn4A	d.166.1.1	3.90.176, 3.90.175, 3.90.210	ADPRT toxin	1	49.5
A0QLI5_651-725	75	0.21	0.088	1qs1A	d.166.1.1, d.166.1.1	3.90.176.10, 3.90.176.10	ADPRT toxin	1	43.5
A0QLI5_1-800	800	0.013	1E-16	2a78B	d.166.1.1	3.90.176.10	ADPRT toxin	1	42.67
A0QLI5_676-725	50	1	0.093	1giqA	d.166.1.1, d.166.1.1	3.90.176.10, 3.90.176.10	ADPRT toxin	1	37
A0QLI5_751-800	50	0.032	0.006	1gxyA	d.166.1.3	3.90.176.10	ADPRT toxin	1	35.25
A0QLI5_701-750	50	6.7	1.1	2gwlA	d.166.1.1	3.90.176, 3.90.175, 3.90.210	ADPRT toxin	1	24
A0QLI5_776-825	50	1.3	0.5	3bw8A	d.166.1.1	3.90.176.10	ADPRT toxin	1	23
A0QLI5_784-825	42	3.3	0.79	3bw8A	d.166.1.1	3.90.176.10	ADPRT toxin	1	20
A0QLI5_651-700	50	10	1.1	1qs1A	d.166.1.1, d.166.1.1	3.90.176.10, 3.90.176.10	ADPRT toxin	1	11.5
A0QLI5_726-775	50	1.9	2.1	1qs1A	d.166.1.1, d.166.1.1	3.90.176.10, 3.90.176.10	ADPRT toxin	1	10.75
A0QLI5_601-700	100	3.7	0.064	1r45A	d.166.1.1	3.90.176.10	ADPRT toxin	1	10.5
A0QLI5_145-365	221	10	0.013	3g67A	a.7.4, a.238.1	1.20.58	signaling/chemoreceptor	2	93
A0QLI5_150-440	291	1.8	0.063	2btzA	a.29.5, d.122.1	1.20.140.20, 3.30.565.10	transferase	2	91.25
A0QLI5_95-440	346	2.9	0.008	3g67A	a.7.4, a.238.1	1.20.58	signaling/chemoreceptor	2	88.5
A0QLI5_95-350	256	10	0.004	3g67A	a.7.4, a.238.1	1.20.58	signaling/chemoreceptor	2	82.75
A0QLI5_151-350	200	5.7	0.015	2efrA	a.114.1, a.114.1	1.20.1000	tropomyosin / leucine zipper	2	77.75
A0QLI5_150-350	201	6.4	0.015	2efrA	a.114.1, a.114.1	1.20.1000	tropomyosin / leucine zipper	2	76.25
A0QLI5_134-382	249	10	0.011	1quuA	a.7.1.1, a.7.1.1	1.20.58.60, 1.20.58.60	spectrin	2	75.75
A0QLI5_130-350	221	10	0.011	2ch7A	h.4.5.1	?	chemotaxis	2	74.25
A0QLI5_1-500	500	4	0.28	2ch7A	h.4.5.1	?	chemotaxis	2	63.25
A0QLI5_130-440	311	2.4	0.038	3g67A	a.7.4, a.238.1	1.20.58	signaling/chemoreceptor	2	62
A0QLI5_190-440	251	1.5	0.024	2fxmA	h.1.26.1	?	contractile protein	2	59.5
A0QLI5_101-250	150	0.51	0.6	2vrzA	a.238.1	1.20.1270	viral protein	2	59
A0QLI5_251-350	100	7.7	0.21	2elbA	a.238.1.1, b.55.1.1	1.20.1272, 2.30.29	bar-ph protein binding	2	55.5
A0QLI5_151-225	75	10	1.6	2vrzA	a.238.1	1.20.1270	viral protein	2	54.75
A0QLI5_1-87	87	1.7	2	2vrzA	a.238.1	1.20.1270	viral protein	2	54.5
A0QLI5_1-490	490	4.3	0.29	3g67A	a.7.4, a.238.1	1.20.58	signaling/chemoreceptor	2	53
A0QLI5_190-350	161	4.4	0.048	1kmiZ	h.4.11.1	1.20.5.590, 1.10.287.500	signaling protein	2	52.5

A0QLI5_201-450	250	1.7	0.005	3g67A	a.7.4, a.238.1	1.20.58	signaling/chemoreceptor	2	51.25
A0QLI5_151-250	100	1.2	0.88	2vrzA	a.238.1	1.20.1270	viral protein	2	50.75
A0QLI5_1-476	476	4.2	0.021	3g67A	a.7.4, a.238.1	1.20.58	signaling/chemoreceptor	2	47.5
A0QLI5_186-291	106	1.1	0.1	2ch7A	h.4.5.1	?	chemotaxis	2	42
A0QLI5_179-286	108	0.92	0.007	1sj8A	a.215.1.1, a.216.1.1	1.20.1420.10	talin	2	39.5
A0QLI5_201-400	200	4.7	0.11	1hciA	a.7.1.1, a.7.1.1, a.7.1.1	1.20.58.60, 1.20.58.60, 1.20.58.60	triple-helix coiled coil	2	37
A0QLI5_201-300	100	2.6	0.34	3fyqA	a.24.9	1.20.120	cell adhesion	2	29.5
A0QLI5_201-350	150	4.2	0.063	2ch7A	h.4.5.1	?	chemotaxis	2	25.25
A0QLI5_251-550	300	1.6	0.46	2ch7A	h.4.5.1	?	chemotaxis	2	22.25
A0QLI5_226-275	50	10	1.8	3iqtA	a.24.10	1.20.120	transferase	2	10.5
A0QLI5_169-695	527	3	1E-33	3hkzC	e.29.1	?	RNA polymerase	3	74.67
A0QLI5_190-675	486	2.6	2E-30	3hkzC	e.29.1	?	RNA polymerase	3	73
A0QLI5_150-675	526	2.8	1E-37	3hkzC	e.29.1	?	RNA polymerase	3	72
A0QLI5_96-674	579	3.9	1E-26	3hkzC	e.29.1	?	RNA polymerase	3	71
A0QLI5_95-675	581	3.8	4E-30	1i3qA	e.29.1.2	?	RNA polymerase	3	51
A0QLI5_1-675	675	4.7	6E-25	3h0gA	e.29.1	?	RNA polymerase	3	48.33
A0QLI5_238-783	546	3.4	1E-26	3h0gA	e.29.1	?	RNA polymerase	3	46.33
A0QLI5_1-450	450	4.1	0.024	2g38B	a.25.4.2	?	PPE [from PE/PPE complex]	4	71.25
A0QLI5_1-350	350	10	0.007	2g38B	a.25.4.2	?	PPE [from PE/PPE complex]	4	70.75
A0QLI5_1-237	237	9.1	2.2	2g38B	a.25.4.2	?	PPE [from PE/PPE complex]	4	55.5
A0QLI5_1-250	250	10	1.8	1wa8A	a.25.3.1	?	CFP-10	4	54.5
A0QLI5_1-315	315	10	0.005	2g38B	a.25.4.2	?	PPE [from PE/PPE complex]	4	53.75
A0QLI5_1-400	400	10	0.004	2g38B	a.25.4.2	?	PPE [from PE/PPE complex]	4	53.75
A0QLI5_1-300	300	10	0.012	2g38B	a.25.4.2	?	PPE [from PE/PPE complex]	4	53.25
A0QLI5_1-150	150	0.86	5.3	1wa8B	a.25.3.1	?	ESAT-6	5	64.5
A0QLI5_1-144	144	0.39	0.64	1wa8B	a.25.3.1	?	ESAT-6	5	64.5
A0QLI5_1-100	100	1.6	1.7	1wa8B	a.25.3.1	?	ESAT-6	5	63.75
A0QLI5_1-144	144	0.83	0.99	1wa8B	a.25.3.1	?	ESAT-6	5	63
A0QLI5_1-133	133	0.8	1.2	1wa8B	a.25.3.1	?	ESAT-6	5	62.75
A0QLI5_1-200	200	2	1.8	1wa8B	a.25.3.1	?	ESAT-6	5	60
A0QLI5_1-185	185	1.3	2.4	1wa8B	a.25.3.1	?	ESAT-6	5	58.75
A0QLI5_1-650	650	5.1	6E-31	1wa8B	a.25.3.1	?	ESAT-6	5	48.33
A0QLI5_1-750	750	5.2	4E-08	1wa8B	a.25.3.1	?	ESAT-6	5	47.67
A0QLI5_1-735	735	5.1	0.00004	1wa8B	a.25.3.1	?	ESAT-6	5	47.67
A0QLI5_1-700	700	5.1	1E-21	1wa8B	a.25.3.1	?	ESAT-6	5	47.67
A0QLI5_1-639	639	5.2	1E-29	1wa8B	a.25.3.1	?	ESAT-6	5	47.67
A0QLI5_1-600	600	4.8	3E-18	1wa8B	a.25.3.1	?	ESAT-6	5	46.67
A0QLI5_1-75	75	1.7		1wa8B	a.25.3.1	?	ESAT-6	5	44.25
A0QLI5_1-530	530	4.3	0.049	1wa8B	a.25.3.1	?	ESAT-6	5	36.25
A0QLI5_1-550	550	4.5	9E-15	1wa8B	a.25.3.1	?	ESAT-6	5	35.75
A0QLI5_1-50	50	3.6		1wa8B	a.25.3.1	?	ESAT-6	5	10.25
A0QLI5_251-300	50	10	0.19	2npsD	f.5.1	?	transport protein		31
A0QLI5_351-750	400	2.4	1.5	2pneA	?	?	antifreeze protein		24.67
A0QLI5_51-150	100	1.7	0.68	1colA	f.1.1.1	1.10.490.30	antibacterial protein		23.5
A0QLI5_350-440	91	0.63	0.089	1nlrA	b.29.1.11	2.60.120.180	endoglucanase		19.75
A0QLI5_301-400	100	1.9	0.7	1s0hA	a.1.1.2	1.10.490.10	oxygen storage/transport		19.5
A0QLI5_316-407	92	1.6	1.4	1rlrA	a.98.1.1, c.7.1.2	?	oxidoreductase		18.5
A0QLI5_401-500	100	10	0.15	2pneA	?	?	antifreeze protein		18.5
A0QLI5_408-727	320	4.9	8	1m2vB	a.71.2.1, b.2.8.1, c.62.1.2	3.40.50.410	protein transport		18.25
A0QLI5_201-275	75	2.2	0.16	3jvjA	a.29.2	1.20.920	signaling protein		17.5
A0QLI5_22-103	82	3.1	3.7	1v53A	c.77.1.1	3.40.718.10	oxidoreductase		17.25
A0QLI5_351-450	100	0.69	0.057	2v4jC	d.203.1.1	3.30.1420.10, 1.10.10.370	oxidoreductase		17
A0QLI5_423-514	92	10	0.002	3bogA	?	?	antifreeze protein		16.75
A0QLI5_88-168	81	4.5	2.6	2d4vA	c.77.1	3.40.718.10	oxidoreductase		16.5

A0QLI5_296-422	127	0.58	0.41	1fhiA	d.13.1.1	3.30.428.10	nucleotide-binding protein		16
A0QLI5_301-375	75	2.2	1.3	2o2hA	c.69.1	3.40.50.1820	hydrolase		16
A0QLI5_351-425	75	0.54	0.09	2pl5A	c.69.1.40	?	transferase		15.75
A0QLI5_95-155	71	4.1		1zzhA	a.3.1, a.3.1	1.10.760	oxidoreductase		14
A0QLI5_51-125	75	1.1	1.1	1xg6A	b.42.4	2.80.10.50	hydrolase inhibitor		14
A0QLI5_101-200	100	10	1.2	2wroU	?	?	ribosome		13.5
A0QLI5_501-600	100	10	0.037	1jj2A	b.34.5.3, b.40.4.5	2.30.30.30, 2.40.50.140, 4.10.950.10	ribosome		13.25
A0QLI5_51-100	50	10		1ajrA	c.67.1.1	3.90.1150.10, 3.40.640.10	aminotransferase		12.25
A0QLI5_26-75	50	3.2		1isoA	c.77.1.1	3.40.718.10	oxidoreductase		12
A0QLI5_176-225	50	5.6	2.4	1p7tA	c.1.13.1	1.10.1860.10, 2.170.170.11, 3.20.20.360	lyase		11.5
A0QLI5_126-175	50	10	1.2	3do6A	c.37.1	3.40.50.300, 3.30.1510.10	ligase		11.5
A0QLI5_376-425	50	1.3	0.079	2pl5A	c.69.1.40	?	transferase		11.25
A0QLI5_351-400	50	6		3a21A	c.1.8, b.42.2, b.71.1, b.18.1	3.20.20, 2.80.10, 2.60.40, 2.60.120	hydrolase		11.25
A0QLI5_477-530	54	10	0.5	2an1A	e.52.1	?	transferase		11
A0QLI5_104-178	75	10	3.5	1r5eA	a.8.8.1	?	protein binding		10.75
A0QLI5_401-450	50	10		1f03A	d.120.1.1	3.10.120.10	electron transport		10.75
A0QLI5_326-375	50	2.2	1.1	1ipaA	c.116.1.1, d.79.3.3	3.30.1330.30, 3.40.1280.10,	transferase		10.25
A0QLI5_476-525	50	10	1.1	2an1A	e.52.1	?	transferase		10.25
A0QLI5_626-675	50	10	2	1ea7A	c.41.1.1	3.40.50.200	hydrolase		10.25
A0QLI5_301-350	50	8	3.7	2fztA	a.46.3.1	?	structural genomics		10
A0QLI5_151-200	50	10	0.56	1onfA	c.3.1.5, c.3.1.5, d.87.1.1	3.50.50.60, 3.50.50.60, 3.30.390.30	oxidoreductase		10

EFV toxin

target name	len	blast	pdb-psi	pdb	scop	cath	interpret	rank	3djury
Q838U8	487	2E-09	1E-25	2j3zA	d.166.1.1	3.90.176, 3.90.175, 3.90.210	ADPRT toxin	1	158.12
Q838U8_1-450	450	0.037	2E-18	1giqA	d.166.1.1, d.166.1.1	3.90.176.10, 3.90.176.10	ADPRT toxin	1	136
Q838U8_251-487	237	8E-08	3E-36	1gxyA	d.166.1.3	3.90.176.10	ADPRT toxin	1	124.75
Q838U8_262-487	226	6E-08	2E-34	1giqA	d.166.1.1, d.166.1.1	3.90.176.10, 3.90.176.10	ADPRT toxin	1	124
Q838U8_301-487	187	2E-08	5E-30	1giqA	d.166.1.1, d.166.1.1	3.90.176.10, 3.90.176.10	ADPRT toxin	1	122.75
Q838U8_313-487	175	1E-08	2E-30	2wn4A	d.166.1.1	3.90.176, 3.90.175, 3.90.210	ADPRT toxin	1	115.75
Q838U8_328-478	151	7E-08	5E-25	1giqA	d.166.1.1, d.166.1.1	3.90.176.10, 3.90.176.10	ADPRT toxin	1	100.75
Q838U8_1-417	417	0.22	6E-13	2wn4A	d.166.1.1	3.90.176, 3.90.175, 3.90.210	ADPRT toxin	1	98
Q838U8_301-450	150	0.003	3E-23	2wn4A	d.166.1.1	3.90.176, 3.90.175, 3.90.210	ADPRT toxin	1	97.75
Q838U8_201-450	250	0.017	1E-25	2wn4A	d.166.1.1	3.90.176, 3.90.175, 3.90.210	ADPRT toxin	1	93.25
Q838U8_1-400	400	0.39	4E-12	2wn4A	d.166.1.1	3.90.176, 3.90.175, 3.90.210	ADPRT toxin	1	91.25
Q838U8_351-487	137	0.000005	9E-27	2wn4A	d.166.1.1	3.90.176, 3.90.175, 3.90.210	ADPRT toxin	1	89.75
Q838U8_301-400	100	0.045	9E-22	2wn4A	d.166.1.1	3.90.176, 3.90.175, 3.90.210	ADPRT toxin	1	68.5
Q838U8_384-487	104	0.002	1E-26	1qs1A	d.166.1.1, d.166.1.1	3.90.176.10, 3.90.176.10	ADPRT toxin	1	68.33
Q838U8_211-407	197	0.14	4E-14	2wn4A	d.166.1.1	3.90.176, 3.90.175, 3.90.210	ADPRT toxin	1	66.5
Q838U8_201-300	200	0.2	1E-13	2wn4A	d.166.1.1	3.90.176, 3.90.175, 3.90.210	ADPRT toxin	1	63.5
Q838U8_351-450	100	0.085	0.11	1ojqA	d.166.1.1	3.90.176.10	ADPRT toxin	1	61.5
Q838U8_384-477	94	0.01	6E-10	1qs1A	d.166.1.1, d.166.1.1	3.90.176.10, 3.90.176.10	ADPRT toxin	1	58
Q838U8_401-487	87	0.002	0.0003	2wn4A	d.166.1.1	3.90.176, 3.90.175, 3.90.210	ADPRT toxin	1	55
Q838U8_401-475	75	0.011	0.007	3buzA	d.166.1.1, d.166.1.1	3.90.176.10, 3.90.176.10	ADPRT toxin	1	50.75
Q838U8_351-425	75	0.081	0.097	3bw8A	d.166.1.1	3.90.176.10	ADPRT toxin	1	48.75
Q838U8_324-383	60	0.13	0.057	1giqA	d.166.1.1, d.166.1.1	3.90.176.10, 3.90.176.10	ADPRT toxin	1	47.33
Q838U8_418-487	70	0.002	0.001	2wn4A	d.166.1.1	3.90.176, 3.90.175, 3.90.210	ADPRT toxin	1	43.5
Q838U8_328-383	56	0.81	0.095	2wn4A	d.166.1.1	3.90.176, 3.90.175, 3.90.210	ADPRT toxin	1	41.75
Q838U8_301-375	75	0.12	0.079	2j3vA	d.166.1.1	3.90.176, 3.90.175, 3.90.210	ADPRT toxin	1	40.75
Q838U8_376-425	50	9		1gxyA	d.166.1.3	3.90.176.10	ADPRT toxin	1	37.5
Q838U8_351-400	50	2.3	1.3	1giqA	d.166.1.1, d.166.1.1	3.90.176.10, 3.90.176.10	ADPRT toxin	1	37.5
Q838U8_326-375	50	2.6	0.98	1giqA	d.166.1.1, d.166.1.1	3.90.176.10, 3.90.176.10	ADPRT toxin	1	36.75
Q838U8_426-475	50	0.013	0.012	1qs1A	d.166.1.1, d.166.1.1	3.90.176.10, 3.90.176.10	ADPRT toxin	1	32.75
Q838U8_401-450	50	0.43	0.33	3bw8A	d.166.1.1	3.90.176.10	ADPRT toxin	1	22.75
Q838U8_251-350	100	1.5	0.12	2j3vA	d.166.1.1	3.90.176, 3.90.175, 3.90.210	ADPRT toxin	1	11.5
Q838U8_1-310	310	0.31	0.5	1mroB	a.89.1.1, d.58.31.2	3.30.70.470, 1.20.840.10	methanogenesis	2	67.25
Q838U8_1-312	312	0.32	0.68	1mroB	a.89.1.1, d.58.31.2	3.30.70.470, 1.20.840.10	methanogenesis	2	57
Q838U8_1-238	238	0.14	1.2	1u4qA	a.7.1.1, a.7.1.1, a.7.1.1	1.20.58.60, 1.20.58.60, 1.20.58.60	spectrin	2	49.33
Q838U8_1-300	300	0.3	0.28	1sumB	a.7.12.1	1.20.58.220, 1.20.58.220	transport protein	2	36.75
Q838U8_1-207	207	0.16	1.4	3hr0A	a.47.1	1.20.58.240	transport protein	2	33.25
Q838U8_1-210	210	0.18	1.2	3hr0A	a.47.1	1.20.58.240	transport protein	2	32.5
Q838U8_1-350	350	0.35	1.2	1fioA	a.47.2.1	1.20.58.70	membrane protein	2	32.5
Q838U8_1-150	150	0.31	1.5	2wrgI	a.238.1	1.20.1270	viral protein	2	28.5
Q838U8_1-250	250	0.19	0.25	1fioA	a.47.2.1	1.20.58.70	membrane protein	2	26.75
Q838U8_1-100	100	4	1.2	2qupA	a.24.23	1.20.120.490	structural genomics	2	25
Q838U8_101-200	100	1.1	0.26	1f68A	a.29.2.1	1.20.920.10	transferase	2	23.25
Q838U8_1-75	75	2.5	0.6	2jq9A	a.7.14.1	1.20.58	protein transport	2	17.25
Q838U8_101-175	75	2.7	1.6	3g0lA	a.29.2	1.20.920	transcription	2	13.25
Q838U8_1-146	146	0.29	0.16	3fewX	f.1.1	1.10.490	immune system		41.5
Q838U8_91-308	218	0.26	0.53	1chmA	c.55.2.1, d.127.1.1	3.40.350.10, 3.90.230.10	creatinase		33.25
Q838U8_1-200	200	0.17	0.85	1colA	f.1.1.1	1.10.490.30	antibacterial protein		28.5
Q838U8_1-144	144	0.33	0.13	3fewX	f.1.1	1.10.490	immune system		27
Q838U8_201-300	100	0.42	0.33	1q1rA	c.3.1.5, c.3.1.5, d.87.1.1	3.50.50.60, 3.50.50.60, 3.30.390.30	oxidoreductase		24
Q838U8_145-261	117	0.072	0.1	1tpvA	c.1.1.1	3.20.20.70	isomerase(intramolecular oxidoreductase)		23.75
Q838U8_192-300	109	0.34	0.069	3fg2P	c.3.1, d.87.1, c.4.1	3.50.50	oxidoreductase		23.75
Q838U8_1-137	137	0.21	0.39	1qvrA	a.174.1.1, c.37.1.20, c.37.1.2	3.40.50.300, 3.40.50.300, 1.10.8.60	chaperone		23.5
Q838U8_132-300	169	0.16	0.037	1sqbB	d.185.1.1, d.185.1.1	3.30.830.10, 3.30.830.10	oxidoreductase		22.75
Q838U8_147-268	122	0.099	0.091	1lyxA	c.1.1.1	3.20.20.70	isomerase		22
Q838U8_101-250	150	0.12	0.32	2hl7A	?	?	oxidoreductase		21.75
Q838U8_138-300	163	0.16	0.82	3cwbB	d.185.1, d.185.1	3.30.830.10, 3.30.830.10	oxidoreductase		21.5
Q838U8_251-300	50	9.6	0.28	3fybA	?	?	structural genomics		21

Q838U8_151-200	50	3.4	2.2	2e7uA	c.67.1	3.40.640	isomerase		20.5
Q838U8_51-125	75	0.13	3.3	2wltA	c.88.1	?	hydrolase		17.5
Q838U8_201-275	75	0.87	4.5	1bcj1	d.169.1.1, h.1.1.1	3.10.100.10	lectin		14.5
Q838U8_151-350	200	0.24	0.11	1p4wA	a.4.6.2	1.10.10.10	dna binding protein		13.75
Q838U8_151-225	75	0.054	0.075	2rnjA	a.4.6	1.10.10.10	transcription		13.75
Q838U8_151-250	100	0.07	0.18	2q0oA	d.110.5, a.4.6	3.30.450	transcription		13.75
Q838U8_208-300	117	0.47	0.33	1litA	d.169.1.1	3.10.100.10	sugar binding		13.25
Q838U8_26-90	65	5.1	0.72	2kcdA	?	?	structural genomics		12.75
Q838U8_26-75	50	10		1txdA	a.87.1.1, b.55.1.1	2.30.29.30	signaling protein		12.25
Q838U8_176-225	50	1.7	1.3	2ox9A	d.169.1	3.10.100.10	sugar binding		12.25
Q838U8_269-327	59	4	0.99	1f9dA	a.102.1.2	1.50.10.10, 2.170.160.10, 4.10.870.10	hydrolase		12
Q838U8_201-350	150	0.91	0.46	2kftA	g.50.1	3.30.40	transcription/protein binding		11.5
Q838U8_301-350	50	1.4	0.78	1b8xA	a.45.1.1, c.47.1.5	3.40.30.10, 1.20.1050.10, 4.10.770.10	signal protein		11.25
Q838U8_76-125	50	4.7	3.1	2hpoA	d.92.1, b.98.1	3.40, 3.90.1240	hydrolase		11
Q838U8_126-175	50	3.6	1.5	2dvwB	c.37.1	3.40.50, 3.40.850, 3.40.1140, 1.10.8	cell cycle/protein-binding		10.75
Q838U8_51-100	50	2.7	1.2	1toaA	c.92.2.2	3.40.50.1980, 3.40.50.1980	binding protein		10.25
Q838U8_101-150	50	5.2	3.7	1cm5A	c.7.1.1	3.20.70.20	transferase		10

TccC5

target name	len	blast	pdb-psi	pdb	scop	cath	interpret	rank	3djury
Q7N7Y7_1-700	700	1.8	1.1	1fwxA	b.6.1.4, b.69.3.1	2.130.10.10, 2.60.40.420	oxidoreductase	1	123
Q7N7Y7_1-800	800	2.3	1.4	2iwfA	b.69.3, b.6.1	2.130.10.10, 2.60.40.420	oxidoreductase	1	113.25
Q7N7Y7_1-550	550	1.5	2.3	1fwxA	b.6.1.4, b.69.3.1	2.130.10.10, 2.60.40.420	oxidoreductase	1	101
Q7N7Y7_1-500	500	1.2	0.68	2iwfA	b.69.3, b.6.1	2.130.10.10, 2.60.40.420	oxidoreductase	1	99.25
Q7N7Y7_1-400	400	1	0.26	1gotB	b.69.4.1	2.130.10.10	complex (gtp-binding/transducer)	1	94.75
Q7N7Y7_1-583	583	1.3	2.1	2iwfA	b.69.3, b.6.1	2.130.10.10, 2.60.40.420	oxidoreductase	1	92.25
Q7N7Y7_1-900	900	0.44	0.82	1fwxA	b.6.1.4, b.69.3.1	2.130.10.10, 2.60.40.420	oxidoreductase	1	88
Q7N7Y7_1-450	450	1.3	1.3	2iwfA	b.69.3, b.6.1	2.130.10.10, 2.60.40.420	oxidoreductase	1	85.75
Q7N7Y7_1-350	350	0.87	0.71	2iwfA	b.69.3, b.6.1	2.130.10.10, 2.60.40.420	oxidoreductase	1	81.5
Q7N7Y7_1-600	600	1.4	0.6	1fwxA	b.6.1.4, b.69.3.1	2.130.10.10, 2.60.40.420	oxidoreductase	1	77
Q7N7Y7_201-450	250	0.96	2.3	2z2pA	b.68.9	2.120.10	lyase	2	94.25
Q7N7Y7_251-550	300	0.73	0.19	2fp8A	b.68.6	2.120.10	lyase	2	78.5
Q7N7Y7_201-350	150	0.45	0.33	3e5zA	b.68.6	2.120.10	lyase	2	56
Q7N7Y7_401-800	400	1.2	0.69	2z2pA	b.68.9	2.120.10	lyase	2	51
Q7N7Y7_401-850	450	0.22	0.23	2z2pA	b.68.9	2.120.10	lyase	2	50.75
Q7N7Y7_701-938	238	0.089	0.13	1r45A	d.166.1.1	3.90.176.10	ADPRT toxin	3	92
Q7N7Y7_730-938	209	0.09	0.004	1r45A	d.166.1.1	3.90.176.10	ADPRT toxin	3	82.5
Q7N7Y7_651-938	288	0.11	0.036	1r45A	d.166.1.1	3.90.176.10	ADPRT toxin	3	80.25
Q7N7Y7_701-850	150	0.046	0.088	1giqA	d.166.1.1, d.166.1.1	3.90.176.10, 3.90.176.10	ADPRT toxin	3	79.75
Q7N7Y7_751-938	188	0.19	0.007	2a78B	d.166.1.1	3.90.176.10	ADPRT toxin	3	52.5
Q7N7Y7_773-886	144	1	0.012	1qs1A	d.166.1.1, d.166.1.1	3.90.176.10, 3.90.176.10	ADPRT toxin	3	49
Q7N7Y7_584-938	355	0.098	0.45	1ojqA	d.166.1.1	3.90.176.10	ADPRT toxin	3	48.67
Q7N7Y7_701-800	100	0.61	0.16	2wn4A	d.166.1.1	3.90.176, 3.90.175, 3.90.210	ADPRT toxin	3	46.25
Q7N7Y7_701-775	75	0.66	0.59	2wn4A	d.166.1.1	3.90.176, 3.90.175, 3.90.210	ADPRT toxin	3	42.5
Q7N7Y7_751-850	100	0.075	0.16	2a78B	d.166.1.1	3.90.176.10	ADPRT toxin	3	40.25
Q7N7Y7_756-864	109	0.13	0.3	2gwlA	d.166.1.1	3.90.176, 3.90.175, 3.90.210	ADPRT toxin	3	37.25
Q7N7Y7_801-938	138	0.77	0.11	2gwlA	d.166.1.1	3.90.176, 3.90.175, 3.90.210	ADPRT toxin	3	35.5
Q7N7Y7_726-775	50	2.1	0.67	2wn4A	d.166.1.1	3.90.176, 3.90.175, 3.90.210	ADPRT toxin	3	34.75
Q7N7Y7_801-900	100	0.45	0.55	2wn4A	d.166.1.1	3.90.176, 3.90.175, 3.90.210	ADPRT toxin	3	27.75
Q7N7Y7_751-825	75	0.095	0.12	1r45A	d.166.1.1	3.90.176.10	ADPRT toxin	3	27.75
Q7N7Y7_601-938	338	0.15	0.73	2wn4A	d.166.1.1	3.90.176, 3.90.175, 3.90.210	ADPRT toxin	3	16
Q7N7Y7_801-850	50	10	6.2	2wn4A	d.166.1.1	3.90.176, 3.90.175, 3.90.210	ADPRT toxin	3	11
Q7N7Y7_865-918	54	0.98	0.094	3bw8A	d.166.1	3.90.176.10	ADPRT toxin	3	10.5
Q7N7Y7_1-750	750	2.1	1.2	2fkjA	b.76.1	3.90.930	OspA	4	83
Q7N7Y7_1-341	341	0.85	1.6	2fkjA	b.76.1	3.90.930	OspA	4	80.25
Q7N7Y7_301-650	350	6.5	0.79	1fj1E	b.76.1	3.90.930	OspA	4	77
Q7N7Y7_342-729	388	1.1	8.4	2hkdA	b.76.1	3.90.930	OspA	4	74.5
Q7N7Y7_1-850	850	0.41	2.2	2fkjA	b.76.1	3.90.930	OspA	4	73.25
Q7N7Y7_1-300	300	3.3	0.22	1fj1E	b.76.1	3.90.930	OspA	4	71
Q7N7Y7_101-250	150	4.8	0.83	2i5vO	b.76.1	3.90.930	OspA	4	70.25
Q7N7Y7_401-600	200	1.5		2oy7A	b.76.1	3.90.930	OspA	4	70
Q7N7Y7_151-350	200	0.51	2.3	2fkjA	b.76.1	3.90.930	OspA	4	69.25
Q7N7Y7_1-250	250	2.5	1.7	2oy7A	b.76.1	3.90.930	OspA	4	68.75
Q7N7Y7_301-500	200	1.8	0.031	2i5vO	b.76.1	3.90.930	OspA	4	63.5
Q7N7Y7_1-650	650	1.7	0.74	2hkdA	b.76.1	3.90.930	OspA	4	63
Q7N7Y7_351-750	400	1.1	8.8	2hkdA	b.76.1	3.90.930	OspA	4	63
Q7N7Y7_151-250	100	3.8	0.62	1fj1E	b.76.1	3.90.930	OspA	4	62.75
Q7N7Y7_451-650	200	0.88	1.8	2ol6O	b.76.1	3.90.930	OspA	4	60.5
Q7N7Y7_401-650	250	1.6	0.63	1fj1E	b.76.1	3.90.930	OspA	4	60.25

Q7N7Y7_1-200	200	2.3	0.16	1fj1E	b.76.1	3.90.930	OspA	4	58.5
Q7N7Y7_201-400	200	0.7	1.2	2oy5O	b.76.1	3.90.930	OspA	4	56.5
Q7N7Y7_201-300	100	5.2	1.6	2oy8A	b.76.1	3.90.930	OspA	4	53.67
Q7N7Y7_401-550	150	5.6	0.17	2oy8A	b.76.1	3.90.930	OspA	4	51.5
Q7N7Y7_501-800	300	0.82	1.2	2i5vO	b.76.1	3.90.930	OspA	4	46.75
Q7N7Y7_101-200	100	2.4	0.81	2fkgA	b.76.1	3.90.930	OspA	4	43
Q7N7Y7_176-266	91	3.6	0.93	2fkjA	b.76.1	3.90.930	OspA	4	41.5
Q7N7Y7_1-100	100	1.2	0.32	2i5vO	b.76.1	3.90.930	OspA	4	41
Q7N7Y7_501-938	438	0.18	0.29	2hkdA	b.76.1	3.90.930	OspA	4	39
Q7N7Y7_251-350	100	0.49	0.053	2fkjA	b.76.1	3.90.930	OspA	4	38.75
Q7N7Y7_78-175	98	0.97	0.94	2hkdA	b.76.1	3.90.930	OspA	4	37.75
Q7N7Y7_301-450	150	2.7	4.5	2oy8A	b.76.1	3.90.930	OspA	4	36.5
Q7N7Y7_401-500	100	2.9	0.9	2hkdA	b.76.1	3.90.930	OspA	4	36
Q7N7Y7_501-650	150	0.48	0.66	2ol6O	b.76.1	3.90.930	OspA	4	35.5
Q7N7Y7_551-938	388	0.18	0.97	1fj1E	b.76.1	3.90.930	OspA	4	35
Q7N7Y7_1-150	150	1.8	0.014	2oy5O	b.76.1	3.90.930	OspA	4	33.25
Q7N7Y7_51-150	100	1.2	0.74	2fkjA	b.76.1	3.90.930	OspA	4	32.5
Q7N7Y7_451-550	100	4.1	0.45	2oy7A	b.76.1	3.90.930	OspA	4	32.25
Q7N7Y7_551-650	100	2.7	0.29	1fj1E	b.76.1	3.90.930	OspA	4	31.25
Q7N7Y7_101-175	75	0.95	0.24	2oy7A	b.76.1	3.90.930	OspA	4	30.5
Q7N7Y7_201-275	75	8.8	4.5	1fj1E	b.76.1	3.90.930	OspA	4	30.5
Q7N7Y7_51-125	75	7.2	3	2i5vO	b.76.1	3.90.930	OspA	4	29.5
Q7N7Y7_451-525	75	3.9	0.34	2i5vO	b.76.1	3.90.930	OspA	4	29
Q7N7Y7_251-325	75	0.69	0.38	2i5vO	b.76.1	3.90.930	OspA	4	28.75
Q7N7Y7_305-434	130	1.4	0.8	2oy5O	b.76.1	3.90.930	OspA	4	28.5
Q7N7Y7_351-425	75	8.4	3.2	2oy8A	b.76.1	3.90.930	OspA	4	28.5
Q7N7Y7_401-475	75	5.6	0.41	2fkjA	b.76.1	3.90.930	OspA	4	26.75
Q7N7Y7_351-450	100	10	0.4	2oy8A	b.76.1	3.90.930	OspA	4	22.75
Q7N7Y7_76-125	50	5.6	4.2	1fj1E	b.76.1	3.90.930	OspA	4	22.5
Q7N7Y7_101-150	50	2.7	3.9	2fkgA	b.76.1	3.90.930	OspA	4	21
Q7N7Y7_151-225	75	4.9	0.26	2oy5O	b.76.1	3.90.930	OspA	4	18.75
Q7N7Y7_301-400	100	1.4	0.75	1fj1E	b.76.1	3.90.930	OspA	4	16.25
Q7N7Y7_551-625	75	1.2	0.88	2i5vO	b.76.1	3.90.930	OspA	4	15.5
Q7N7Y7_251-300	50	6.3	0.44	2hkdA	b.76.1	3.90.930	OspA	4	12
Q7N7Y7_126-175	50	10	0.25	2i5vO	b.76.1	3.90.930	OspA	4	11.5
Q7N7Y7_326-375	50	1.2	1.6	2oy8A	b.76.1	3.90.930	OspA	4	11.5
Q7N7Y7_1-77	77	1.9	0.56	2i5vO	b.76.1	3.90.930	OspA	4	11
Q7N7Y7_201-250	50	8.6	5.7	2oy8A	b.76.1	3.90.930	OspA	4	11
Q7N7Y7_226-275	50	10	3.2	2i5vO	b.76.1	3.90.930	OspA	4	11
Q7N7Y7_151-200	50	9.9	1.8	2i5vO	b.76.1	3.90.930	OspA	4	10.5
Q7N7Y7	938	0.29	3	1nr0A	b.69.4.1, b.69.4.1	2.130.10.10, 2.130.10.10	structural protein	5	70.75
Q7N7Y7_435-572	138	0.97	0.56	2c26A	b.1.3 / b.18.1	2.60.40, 2.60.120	hydrolase	6	52.25
Q7N7Y7_501-575	75	1.4	1.9	2boiA	b.115.1	2.60.120.400	lectin	6	29
Q7N7Y7_601-675	75	0.19	0.3	2ac1A	b.67.2, b.29.1	2.115.10; 2.60.120, 2.70.10	hydrolase	6	24.25
Q7N7Y7_601-700	100	0.19	0.29	1y4wA	b.29.1.19, b.67.2.3	2.60.120.560	hydrolase	6	22.25
Q7N7Y7_601-750	150	0.33	0.28	2ac1A	b.67.2, b.29.1	2.115.10; 2.60.120, 2.70.10	hydrolase	6	14.75
Q7N7Y7_601-850	250	0.11	0.3	1st8A	b.67.2 / b.29.1	2.115.10; 2.60.120, 2.70.10	hydrolase	6	14.75
Q7N7Y7_601-800	200	0.54	0.64	1st8A	b.67.2 / b.29.1	2.115.10; 2.60.120, 2.70.10	hydrolase	6	14.75
Q7N7Y7_601-650	50	4.5	0.62	1st8A	b.67.2 / b.29.1	2.115.10; 2.60.120, 2.70.10	hydrolase	6	10
Q7N7Y7_451-938	488	0.22	1.1	2pu3A	d.4.1	3.90.540, 3.90.75, 3.40.570	hydrolase		40
Q7N7Y7_501-600	100	0.95	2.6	1p30A	b.121.2.2, b.121.2.2	2.170.9.10, 3.90.39.10, 3.90.249	viral protein		34.25

Q7N7Y7_351-400	50	10	1.5	1ewkA	c.93.1.1	3.40.50.2300, 3.40.50.2300	signaling protein		24
Q7N7Y7_626-675	50	0.24	0.51	2nujA	d.38.1.1	3.10.129.10	hydrolase		23.25
Q7N7Y7_627-675	49	0.34	0.54	2nujA	d.38.1.1	3.10.129.10	hydrolase		23.25
Q7N7Y7_851-938	88	0.8	0.12	1ftkA	c.94.1.1	3.40.190.10, 3.40.190.10	membrane protein		19.75
Q7N7Y7_801-875	75	8.3		2hknA	b.34.10	2.30.30	structural protein		16.5
Q7N7Y7_651-725	75	1.3	0.67	1vh0A	c.116.1.3	3.40.1280.10	structural genomics		16
Q7N7Y7_1-75	75	2	0.54	1qgwA	d.184.1.1	3.90.510.10	photosynthesis		15.75
Q7N7Y7_851-925	75	1	0.14	1zcfA	c.88.1	3.40.50.1170, 3.40.50.40	hydrolase		15.25
Q7N7Y7_573-626	54	5.1	3.2	2w4eA	d.113.1	3.90.79	hydrolase		12.75
Q7N7Y7_677-737	61	10	2.9	1sgcA	b.47.1.1	2.40.10.10, 2.40.10.10	hydrolase (serine proteinase)		12.5
Q7N7Y7_301-375	75	1.5	1.5	1pseA	b.34.4.2	2.30.30.50	photosystem		11.75
Q7N7Y7_276-325	50	1.1	0.51	2iovA	d.22.1	2.40.155	luminescent protein		11.75
Q7N7Y7_451-500	50	4	0.72	2zpcA	b.125.1	2.50.20.10	protein transport		11.75
Q7N7Y7_576-625	50	5.3	7.7	2yvmA	d.113.1	3.90.79	hydrolase		11.75
Q7N7Y7_426-475	50	8	5.8	3exwA	b.21.1	2.60.90.10	viral protein		11.5
Q7N7Y7_51-100	50	9.4	2.3	1l0oA	d.122.1.3	3.30.565.10	protein binding		11.5
Q7N7Y7_401-450	50	6.3	1.7	3f9wA	b.85.7	3.90.1410, 2.170.270	transferase		11.5
Q7N7Y7_676-755	80	3.3	0.96	1pfoA	f.9.1.1	3.90.840.10, 3.30.1040.20, 2.60	toxin		11.5
Q7N7Y7_751-800	50	3.5	8.2	1wiaA	d.15.1.1	3.10.20	structural genomics		11.25
Q7N7Y7_826-875	50	10		1si8A	e.5.1.1	?	oxidoreductase		11
Q7N7Y7_551-600	50	1.4	3.5	1g2pA	c.61.1.1	3.40.50.2020	transferase		11
Q7N7Y7_676-725	50	10	2.9	1vh0A	c.116.1.3	3.40.1280.10	structural genomics		10.75
Q7N7Y7_501-550	50	10	4.8	2oqqA	c.23.1	3.40.50	transcription		10.5
Q7N7Y7_651-750	100	1.8	0.93	1iruC	d.153.1.4	3.60.20.10	hydrolase		10.5
Q7N7Y7_476-525	50	10	0.67	2b3tB	e.38.1.1	?	translation		10.25
Q7N7Y7_301-350	50	4.3	2.6	3bbnP	d.27.1	3.30.1320	ribosome		10
Q7N7Y7_651-700	50	3.2	1.2	2gccA	d.10.1.2	3.30.730.10	transcription factor		10

Vis toxin

target name	len	blast	pdb-psi	pdb	scop	cath	interpret	Rank	3djury
A3UNN4	249	0.002	2E-30	1giqA	d.166.1.1, d.166.1.1	3.90.176.10, 3.90.176.10	ADPRT toxin	1	135
A3UNN4_1-200	200	0.008	4E-29	1giqA	d.166.1.1, d.166.1.1	3.90.176.10, 3.90.176.10	ADPRT toxin	1	126.5
A3UNN4_1-150	150	0.11	3E-21	2wn4A	d.166.1.1	3.90.176, 3.90.175, 3.90.210	ADPRT toxin	1	92.5
A3UNN4_101-249	149	0.036	2E-17	2a78B	d.166.1.1	3.90.176.10	ADPRT toxin	1	72.75
A3UNN4_51-150	100	0.15	2E-16	2bovB	d.166.1.1	3.90.176.10	ADPRT toxin	1	70.25
A3UNN4_101-200	100	0.28	6E-11	2wn4A	d.166.1.1	3.90.176, 3.90.175, 3.90.210	ADPRT toxin	1	60.75
A3UNN4_51-125	75	0.74	0.0002	2wn4A	d.166.1.1	3.90.176, 3.90.175, 3.90.210	ADPRT toxin	1	50.75
A3UNN4_1-100	100	1.3	0.024	3buzA	d.166.1.1, d.166.1.1	3.90.176.10, 3.90.176.10	ADPRT toxin	1	49.5
A3UNN4_151-225	75	0.3	0.003	2a78B	d.166.1.1	3.90.176.10	ADPRT toxin	1	44
A3UNN4_101-175	75	0.3	0.04	1qs1A	d.166.1.1, d.166.1.1	3.90.176.10, 3.90.176.10	ADPRT toxin	1	42.5
A3UNN4_151-249	99	0.21	0.002	2bovB	d.166.1.1	3.90.176.10	ADPRT toxin	1	41
A3UNN4_1-75	75	10	0.34	2j3vA	d.166.1.1	3.90.176, 3.90.175, 3.90.210	ADPRT toxin	1	21.25
A3UNN4_201-249	49	3.6	0.18	2volA	b.1.1, b.1.1	2.60.40.10, 2.60.40.10	immune system	2	11.75
